# The insertion and dysregulation of transposable elements in osteosarcoma and their association with patient event-free survival

**DOI:** 10.1038/s41598-021-04208-5

**Published:** 2022-01-10

**Authors:** Chao Wang, Chun Liang

**Affiliations:** Department of Biology, Miami University, Oxford, Ohio 45056 USA

**Keywords:** Cancer, Computational biology and bioinformatics, Biomarkers, Oncology

## Abstract

The dysregulation of transposable elements (TEs) has been explored in a variety of cancers. However, TE activities in osteosarcoma (OS) have not been extensively studied yet. By integrative analysis of RNA-seq, whole-genome sequencing (WGS), and methylation data, we showed aberrant TE activities associated with dysregulations of TEs in OS tumors. Specifically, expression levels of LINE-1 and Alu of different evolutionary ages, as well as subfamilies of SVA and HERV-K, were significantly up-regulated in OS tumors, accompanied by enhanced DNA repair responses. We verified the characteristics of LINE-1 mediated TE insertions, including target site duplication (TSD) length (centered around 15 bp) and preferential insertions into intergenic and AT-rich regions as well as intronic regions of longer genes. By filtering polymorphic TE insertions reported in 1000 genome project (1KGP), besides 148 tumor-specific somatic TE insertions, we found most OS patient-specific TE insertions (3175 out of 3326) are germline insertions, which are associated with genes involved in neuronal processes or with transcription factors important for cancer development. In addition to 68 TE-affected cancer genes, we found recurrent germline TE insertions in 72 non-cancer genes with high frequencies among patients. We also found that +/− 500 bps flanking regions of transcription start sites (TSS) of LINE-1 (young) and Alu showed lower methylation levels in OS tumor samples than controls. Interestingly, by incorporating patient clinical data and focusing on TE activities in OS tumors, our data analysis suggested that higher TE insertions in OS tumors are associated with a longer event-free survival time.

## Introduction

### Overview of osteosarcoma

Osteosarcoma (OS) is a malignant bone tumor that mostly affects young adults (< 20 years old) and a smaller percentage of the elderly (70–85 years)^1^. With low incidence, OS is often classified as an orphan disease^2^. Based on the histological characteristics, conventional OS is the most common type (85%) among all OS cases, with several subtypes identified in conventional OS^3^. The 5-year survival rate for other cancers has been improved in recent years, whereas it has been stagnated for OS (about 60%) since mid-1980s^1^. In clinical settings, about 10–20% of OS patients typically show detectable metastases that commonly occur in lungs (90%), and the 5-year overall survival rate for these patients is only about 20–30%^2^. It is speculated that the remaining 80–90% of patients also have metastases, which, however, are difficult to detect with current diagnostic techniques. Surgery combined with chemotherapy remains an essential treatment for OS patients, where the histological response to preoperative chemotherapy was suggested as the most important prognostic factor to date, with the 5-year event-free survival rate for good responders often at 70–80%^2^. Unfortunately, different subtypes are often mixed in OS tumor tissues, making it harder to evaluate prognostic values of histological classifications^4^. At present, the molecular pathogenesis for different subtypes of the conventional OS is still not well understood^3^. The occurrence of OS has been associated with several rare cancer predisposition syndromes including Li–Fraumeni syndrome (mutations of the TP53 gene), hereditary retinoblastoma (mutations of the RB1 gene), Bloom syndrome (mutations of the RECQL3 gene), Werner syndrome (mutations of the RECQL2 (WRN) gene), and Rothmund–Thomson-syndromes (mutations of the RECQL4 gene)^5^. In addition to these germline predispositions, OS is often characterized by complex karyotypes such as frequent aneuploidy, copy number alterations, and structural aberrations^5^. The high genome instability of many cancer patients has been associated with the phenomenon called chromothripsis^6^ in which a massive genomic rearrangement occurs due to a catastrophic event where chromosomes are fragmented and aberrantly assembled. Another relevant phenomenon is kataegis where the localized hypermutation occurs in the breakage points of chromosomal fragmentation^6^. Both chromothripsis and kataegis have been observed with high frequency in OS genomes^5,6^. Interestingly, chromothripsis has also recently been linked to both somatic and germline TP53 gene mutations in pediatric medulloblastoma and acute myeloid leukemia^3^, and TP53 and RB1 were identified as the only recurrently altered genes in a recently OS study^7^. Apart from those reported mutations, it was shown that over-expression of IGF2 and IRX1 via the hypomethylation of their promoters were involved in the OS-associated metastasis^8^. Dysregulations of micro-RNAs including down-regulation of miR-200b, miR-101, miR-3928, and miR-143 and up-regulation of miR-17–92, miR-574-3p, and miR-542-5p were also implicated in the OS development^8,9^. Obviously, our understanding of the etiology of OS is still limited and the prognostic biomarkers for OS progression are still lacking.

### TEs in human cancers

Increasing amounts of evidence have suggested that activated human transposon elements (TEs) may contribute to genomic instability, a phenomenon widely observed in cancer patients^10^. Based on the insertion mechanism, there are two classes of TEs. Class I is the retrotransposons that use RNA as their intermediate for insertions, including Long Terminal Repeat (LTR) retrotransposons and non-LTR retrotransposons (e.g., Long Interspersed Nuclear Element (LINE), and Short Interspersed Nuclear Element (SINE)). Class II consists of DNA transposons that do not rely on RNA intermediates for insertions^11^. The human genome contains four major groups of retrotransposons (i.e., LINE-1, Alu, SINE-VNTR-Alu (SVA), and human endogenous retroviruses K (HERV-K)) that are currently active, among which LINE-1, a member of LINE and Alu, a member of SINE have been extensively studied. In total, LINE-1 makes up approximately 17% of the human genome while there are more than one million copies of Alu repeats in the human genome^10^. The intact sequence of LINE-1 is about 6 kb long, containing two open reading frames (ORFs), one of which, ORF2 encodes the endonuclease domain and reverse transcriptase domain important for LINE-1 mediated TE insertions (namely insertions of LINE-1, Alu, and SVA)^10^. LINE-1 mediated TE insertions typically end with a 3′ poly(A) tail generated by polyadenylation. HERV-K is a member of LTR retrotransposons that are autonomous TEs, independent of LINE-1 for their insertions^10^. TEs are generally believed to be beneficial for species, where they can help maintain the integrity of centromere and telomere, and they have also been extensively domesticated during the genome evolution^12^ (namely, ORF of TE is co-opted to serve a host function). Most TEs in the human genome are defective due to accumulated mutations^10^. However, it is estimated that about 80–100 LINE-1 elements that are evolutionary young are still fully functional and can generate LINE-1 mediated insertions^13^. Therefore, it is deleterious at the individual organism level if TE activities are not properly regulated^12^.

The dysregulated TE insertions have been analyzed in various cancers. For example, by analyzing whole-genome sequencing (WGS) data in a total of 43 colorectal, prostate, ovarian, multiple myeloma, and glioblastoma cancer samples and their paired normal blood samples, somatic LINE-1, and Alu insertions were more frequently observed in cancers of epithelial cell origin (*e.g*., colorectal, prostate, and ovarian) compared to blood or brain cancers^14^. Similarly, a previous study exclusively focusing on somatic LINE-1 insertions in 244 cancer patients of 12 cancer types identified that 53% of patients had at least one somatic LINE-1 insertion, with colorectal (93% of colorectal cancer patients) and lung cancers (75% of lung cancer patients) being the most frequently affected^15^. Furthermore, a recent study focusing on TE insertions (i.e., LINE-1, Alu, SVA, and HERV) in 202 colorectal tumors showed that the average number of somatic TE insertions is 25 with wide variability among individuals^16^. In terms of the genomic context of TE insertions, the majority of somatic insertions were either found in intergenic regions or intronic regions^15–17^, although somatic LINE-1 insertions were suggested to occur preferentially within the region of DNA hypomethylation^14^. No general effects of LINE-1 insertions on expression levels of affected genes or production of aberrant RNA species (i.e., gene fusion due to 3′ transduction of LINE-1) were found^15^. On the other hand, the study characterizing TE insertions in colorectal tumors mentioned above showed that a higher number of somatic TE insertions was associated with genes of lower expression levels^16^. After accounting for the gene length, no significant enrichment of biological functions for genes with recurrent insertions (i.e., insertions in the same gene occurred in at least two patients) was found^16^. However, a higher number of somatic TE insertions, including LINE-1, Alu, SVA, and HERV was associated with poorer disease-specific survival in colorectal cancer patients^16^. In terms of TE expressions, it has been reported that a significant increase in LINE-1 expression levels was observed in breast invasive carcinoma, head, and neck squamous cell carcinoma, and lung adenocarcinoma when expressions of full-length LINE-1 were considered^17^.

### Regulation of human TEs

Given the mutagenic potential of active TEs, hosts have evolved several ways to regulate their activities. For example, piwi proteins and their associated piwi RNA complexes have been extensively studied for their roles in silencing TE transcriptions^13^. In normal mammalian cells, among others, epigenetic silencing including DNA methylation and histone modification are major mechanisms for regulating TE transcriptions^11^. The interplay between DNA methylation and histone acetylation is critical for the regulation of chromatin conformation, which in turn affects the regulation of TE transcriptions^18^. Furthermore, hosts can regulate TE activities via P53 stress responses triggered by double-stranded breaks that can be generated during TE insertions, resulting in cell cycle arrests or even cell death via apoptosis^13^. Consequently, both P53 mutations and CDKN2A focal deletion were significantly correlated with increased LINE-1 insertions^19^ and P53 mutation was also associated with the enhanced expression of LINE-1 ORF1p protein in lung cancer^10^.

Even though accumulated evidence suggested that dysregulation of TEs plays an important role in the development of different types of cancers, the insertional landscape, activities of TEs, and their aberrant regulations in OS patients have not been fully investigated and characterized yet. Here, we measured transcriptional activities of TEs in OS patients by analyzing RNA-seq data, characterized OS patient-specific TE insertions (namely, both germline and somatic insertions including tumor-specific and normal-specific insertions) using WGS data, explored their dysregulations by analyzing the corresponding DNA methylation data, and finally investigated the prognostic value of TE activities for potential clinical usage in OS patients.

## Results

### Up-regulation of TE expressions in OS tumors

For RNA-Seq analysis, expression levels of 158,859 transcripts, namely 157,679 gene transcript isoforms and 1180 different repeats (including 127 LINE-1 subfamilies, 46 Alu subfamilies, 6 SVA subfamilies, 10 HERV-K subfamilies, and 22 satellite repeats) were obtained as a count table for each sample. In total, 56 count tables were generated. Among them, 52 corresponded to OS tumor samples (i.e., 36 patient’s tumor RNA-seq data from the Therapeutically Applicable Research to Generate Effective Treatments (TARGET) phs000468 study and 16 patient’s tumor RNA-seq data from SRP193919 study), and the remaining 4 were RNA-seq data of normal muscle tissue adjacent to osteosarcoma from the SRP193919 study^20^. For the comparison of sample similarities, 60,430 out of 158,859 rows in all count tables were kept after filtering out rows with low read counts. Based on the similarity of expression profiles, all OS tumor samples were clustered together while 4 adjacent normal muscle tissue samples formed a distinct cluster (see Supplementary Fig. S1a), suggesting that OS tumor samples from both the SRP193919 study and TARGET phs000468 study are comparable.

**Figure 1:**
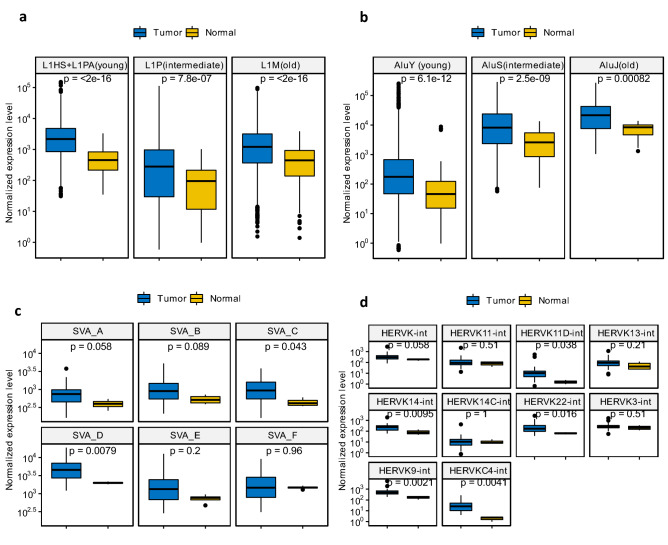
The expression comparisons of TEs (i.e., LINE-1, Alu, SVA, and HERV-K) between OS tumor samples and samples of normal muscle tissue adjacent to osteosarcoma (denoted as normal controls here). (**a**) The expression levels of LINE-1 of different evolutionary ages in OS tumor samples compared with normal controls. (**b**) The expression levels of Alu of different evolutionary ages in OS tumor samples compared with normal controls. (**c**) The expression levels of SVA subfamilies in OS tumor samples compared with normal controls. (**d**) The expression levels of HERV-K subfamilies in OS tumor samples compared with normal controls. (Non-parametric unpaired two-sample Wilcoxon test was used for statistical significance tests between expression levels of TEs in OS tumor samples and normal controls.).

Based on evolutionary ages, 127 LINE-1 subfamilies were grouped into three LINE-1 categories: L1HS + L1PA (young), L1P (intermediate), and L1M (old) whereas 46 Alu subfamilies into three Alu categories: AluY (young), AluS (intermediate), and Alu (old) (see Supplementary Table S1). The expression levels of different categories of LINE-1 and Alu, as well as different subfamilies of SVA and HERV-K, were compared between OS tumor samples and their adjacent normal muscle tissue samples as normal controls. As shown in Fig. 1a,b, all categories of LINE-1 and Alu show significantly higher expressions in OS tumor samples than normal controls (*p* < 0.05 for all cases). The expression comparisons for 6 subfamilies of SVA and 10 subfamilies of HERV-K between OS tumor samples and normal controls were shown in Fig. 1c,d respectively, where 2 SVA subfamilies (i.e., SVA_C, and SVA_D) and 5 HERV-K subfamilies (i.e., HERVK11D-int, HERVK14-int, HERVK22-int, HERVK9-int, and HERVKC4-int) are significantly up-regulated in OS tumor samples (*p* < 0.05). Interestingly, among 22 expressed satellite repeats identified in RNA-seq data, 13 display significantly higher expression levels in OS tumor samples than normal controls (see Supplementary Fig. S1b).

The DNA repair responses that could be induced in the host potentially due to the formation of double-stranded DNA breakages during TE insertions were also examined by measuring expression levels of relevant DNA repair associated genes (see Supplementary Fig. S1c–e and Supplementary Table S2). In terms of 26 genes involved in the homologous recombination, 15 (i.e., XRCC3, XRCC2, TOP3A, RPA3, RAD54L, RAD54B, RAD51D, RAD51, RAD50, POLD3, POLD1, NBN, EME1, BRCA2, and BLM) are significantly up-regulated in OS tumor samples than normal controls (see Supplementary Fig. S1c). Out of 11 genes associated with the non-homologous end joining, 4 (i.e., XRCC4, PRKDC, FEN1, and DCLRE1C) display significantly higher expressions in OS tumor samples than normal controls while DNTT has no detectable expression in normal controls (see Supplementary Fig. S1d). Similarly, out of 14 genes involved in DNA mismatch repair, 10 *(*i.e., RFC5, RFC4, RFC3, RFC1, PCNA, MSH6, MSH3, MSH2, LIG1, and EXO1) show significantly higher expression levels in OS tumor samples than normal controls (see Supplementary Fig. S1e).

### Characterizations of LINE-1 mediated TE insertions

WGS data of 39 OS patients including both tumors and paired blood-derived normal samples were used for TE insertion detections. The normal samples in general have lower sequencing coverages compared with tumor samples (see Supplementary Fig. S2a) based on the result of alignments. Software MELT (Version: 2.1.5, https://melt.igs.umaryland.edu/downloads.php)^21^ and Mobster (Version: 0.2.4, https://jyhehir.github.io/mobster/downloads.html)^22^ were selected for TE insertion detections based on recent benchmarking comparison studies^23,24^. To reduce false-positive calls in TE insertions by either tool, the intersection or consensus of TE insertions identified by both tools for the same sample was used for downstream analyses (see Supplementary Fig. S2b). A total of 39,599 TE insertions were found in OS patients, among which 22,001 including 2940 LINE-1, 18,449 Alu, 598 SVA, and 14 HERV-K were identified in OS tumor samples and 17,598 including 2043 LINE-1, 15,006 Alu, 538 SVA, and 11 HERV-K were identified in normal samples (see Supplementary Fig. S2c). In the 1KGP study, a total of 16,676 LINE-1 mediated TE insertions (including 3059 LINE-1, 12,779 Alu, and 838 SVA) were identified and reported from 2504 individuals of 26 different populations^25^ (see Supplementary Fig. S2d). Obviously, in both cases, LINE-1 and Alu are the most abundant TE insertions detected in human individuals.

**Figure 2:**
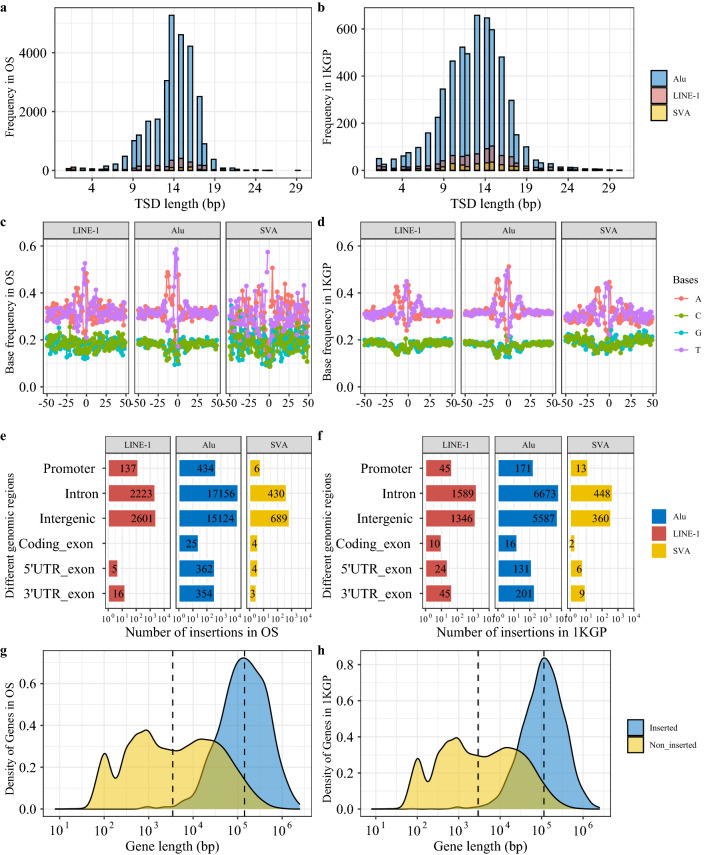
The comparisons for the characteristics of LINE-1 mediated TE insertions identified in OS patients and reported in 1KGP. (**a**) The TSD length distribution of LINE-1, Alu, and SVA insertions identified in OS patients. (**b**) The TSD length distribution of LINE-1, Alu, and SVA insertions reported in 1KGP. (**c**) The nucleotide base compositions within ± 50 bps flanking insertion sites of LINE-1, Alu, and SVA insertions identified in OS patients. (**d**) The nucleotide base compositions within +/− 50 bps flanking insertion sites of LINE-1, Alu, and SVA insertions reported in 1KGP. (**e**) The numbers of LINE-1 mediated TE insertions (i.e., LINE-1, Alu, and SVA) in different genomic regions identified in OS patients. (**f**) The numbers of LINE-1 mediated TE insertions (i.e., LINE-1, Alu, and SVA) in different genomic regions reported in 1KGP. (**g**) The length distribution of genes with LINE-1 mediated TE insertions identified in OS patients versus genes without TE insertions. (**h**) The length distribution of genes with LINE-1 mediated TE insertions reported in 1KGP versus genes without TE insertions. The dashed lines denote relevant median lengths.

Features associated with LINE-1 mediated TE insertions (e.g., target site duplication (TSD) length and insertion preferences) were analyzed. The TSD length distributions of LINE-1 mediated TE insertions identified in OS patients and reported in 1KGP are both concentrated around 15 bps (see Fig. 2a,b). In terms of nucleotide base compositions flanking insertion sites, we found that LINE-1 mediated TE insertions preferentially occur in AT-rich regions of the genome, and this pattern is especially salient for bases close to insertion sites (namely, position 0 on the x-axis) (see Fig. 2c,d). Meanwhile, no discernable pattern of the base composition preference was found for HERV-K insertions identified in OS patients (see Supplementary Fig. S3; 1KPG did not report HERV-K insertions). Most LINE-1 mediated TE insertions identified in OS patients and reported in 1KGP were in intronic and intergenic regions. Specifically, among LINE-1 mediated TE insertions identified in OS patients, 50.06% (19,809 out of 39,574: including 2223 LINE-1, 17,156 Alu, and 430 SVA) are in intronic regions followed by 46.53% (18,414 out of 39,574: including 2601 LINE-1, 15,124 Alu, and 689 SVA) in intergenic regions (see Fig. 2e). Furthermore, out of 39,574 LINE-1 mediated TE insertions identified in OS patients, 624 are in overlapping genomic regions (see Supplementary Table S3). Similarly, 52.23% (8710 out of 16,676: including 1589 LINE-1, 6673 Alu, and 448 SVA) and 43.73% (7293 out of 16,676: including 13,46 LINE-1, 5587 Alu, and 360 SVA) of LINE-1 mediated TE insertions reported in 1KGP are in intronic and intergenic regions respectively (see Fig. 2f), and 345 out of 16,676 insertions were identified in overlapping genomic regions (see Supplementary Table S3). To explore the preference of LINE-1 mediated TE insertions in terms of affected gene lengths, a total of 232,080 annotated transcript isoforms were obtained from hg38 annotation. For the same gene with multiple transcript isoforms annotated in hg38, the longest one was used for the analysis, which resulted in a total of 59,390 transcript isoforms representing each gene. Among these 59,390 genes, about 2.68% (1589) were affected by LINE-1 mediated TE insertions identified in OS patients and about 6.75% (4011) were affected by LINE-1 mediated TE insertions reported by 1KGP. The length distributions of these genes stratified by TE insertion status (i.e., inserted vs non-inserted) indicate that LINE-1 mediated TE insertions are more likely to disrupt longer genes, as shown in Fig. 2g,h, for both insertions identified in OS patients and reported in 1KGP.

The common fragile sites (CFSs) of chromosomes were suggested to be susceptible to sister chromatid exchange, viral integration, deletion, amplification, translocation as well as TE insertions in various cancers^16,19^. To explore the connection between CFS regions and LINE-1 mediated TE insertions identified in OS patients and reported in 1KGP, a total of 124 CFSs were downloaded (https://webs.iiitd.edu.in/raghava/humcfs/^26^). In terms of overlaps between TE insertions and CFSs, the majority of CFSs contain insertions (see Supplementary Fig. S4). Specifically, out of 39,574 LINE-1 mediated TE insertions identified in OS patients, 11,716 (29.61%) occurred within 91.94% of CFSs (114 out of 124). Out of 16,676 LINE-1 mediated TE insertions reported in 1KGP, 5119 (30.7%) occurred within 93.55% of CFSs (116 out of 124). Interestingly, LINE-1 mediated TE insertions reported in 1KGP are relatively spread out across chromosomes, whereas LINE-1 mediated TE insertions identified in OS patients show clear peaks along each chromosome (see Supplementary Fig. S4). This is also reflected by the relatively small proportion of genes that are affected by LINE-1 mediated TE insertions in OS patients compared to those of 1KGP (2.68% of annotated genes for LINE-1 mediated TE insertions in OS patients vs 6.75% of annotated genes in 1KGP) even though more LINE-1 mediated TE insertions were identified in OS patients than those reported in 1KGP (39,574 insertions in OS patients vs 16,676 insertions in 1KGP).

### Identification of OS patient-specific TE insertions

To ascertain the effects of TE insertions on a given disease, it is important to differentiate polymorphic TE insertions^24^ observed in the population from TE insertions that are exclusively occurred in that disease. Therefore, to determine OS patient-specific TE insertions for a given type of TE, a range of values for the distance between insertion sites detected in OS patients and insertion sites reported in 1KGP were used to compare and filter out polymorphic TE insertions. As shown in Supplementary Fig. S5, each bar represents the total number of OS patient-specific TE insertions after filtering out polymorphic TE insertions reported in 1KGP if the insertion distance of the same type of TE (i.e., LINE-1, Alu, or SVA) between OS patients and 1KGP was within the distance specified in the x-axis. Clearly, a distance of 20 bps can effectively reduce the number of potentially OS patient-specific TE insertions from 10,347 (0 bp distance) to 3326 while distances greater than 20 bps have diminishing returns for the elimination of potentially polymorphic TE insertions. Therefore, a distance of 20 bps was selected as the optimal distance threshold to filter out polymorphic TE insertions identified in OS patients using 1KGP insertions as the reference. A total of 3326 OS patient-specific TE insertions were identified after the filtering, of which, 52.77% (1755 out of 3326) were mapped to intergenic regions followed by 44.56% (1482 out of 3326) to introns (see Supplementary Fig. S6a). Out of these OS patient-specific TE insertions, 44 were identified in overlapping genomic regions (see Supplementary Table S3) while most insertions were mapped to unique genomic regions. Among all 3326 OS patient-specific TE insertions, 63.0% (2096 out of 3326) are Alu insertions, and most of them (1703 out of 2096) belong to the AluY (young) category. Among 1108 OS patient-specific LINE-1 insertions, the majority of them (1056 out of 1108) are evolutionary young LINE-1 subfamilies (see Supplementary Fig. S6b–d). It is worth mentioning that all 3326 OS patient-specific TE insertions were visually validated as true cases in Integrative Genomics Viewer (IGV^27^) with the criteria described in the method section.

**Figure 3:**
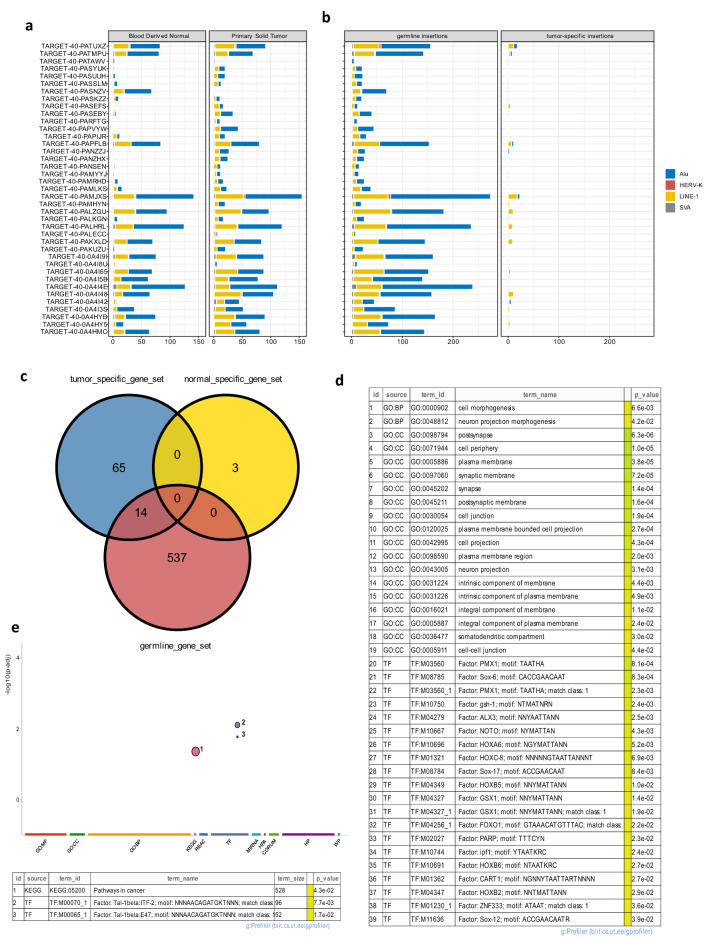
The 3326 OS patient-specific TE insertions were identified after filtering out 16,676 polymorphic TE insertions reported in 1KGP. (**a**) The number of OS patient-specific TE insertions across tumor samples and paired normal samples in 39 OS patients. (**b**) The number of germline TE insertions and tumor-specific somatic TE insertions across 39 OS patients, verified by visual validations via IGV. There are only 3 normal-specific somatic TE insertions (not shown in the figure). (**c**) The number of corresponding genes affected by different insertion types (i.e., germline TE insertions, tumor-specific somatic TE insertions, and normal-specific somatic TE insertions). (**d**) Statistically significant gene ontology terms and relevant transcription factors associated with genes affected by germline TE insertions identified in OS patients. (**e**) Statistically significant KEGG pathway and relevant transcription factors associated with genes affected by tumor-specific somatic TE insertions. (The hypergeometric test implemented in the **g:** Profiler^69^ was used for the statistical enrichment analysis and only annotated genes were used for the statistical domain scope, the default **g**: SCS method was used for multiple testing correction, and the adjusted *p* value < 0.05 was used to select statistically significant terms).

Based on OS patient-specific TE insertions, we further examined TE insertions in each patient (see the method section for details). We observed extremely high heterogeneity in terms of TE insertion frequencies among both tumor samples and their paired normal samples in 39 OS patients (see Fig. 3a). For each patient, after comparing the tumor sample with its paired normal control using a 100-bp window to identify the somatic or germline TE insertions followed by visual validation via IGV, we found that most OS patient-specific TE insertions belong to germline TE insertions (see Fig. 3b). Specifically, among 3326 OS patient-specific TE insertions, 3175 were germline insertions whereas the remaining 151 were somatic insertions. Among these somatic insertions, 148 were tumor-specific and 3 were normal-specific, and we also noticed that most tumor-specific TE insertions are LINE-1 insertions (see Fig. 3b). Out of 3175 germline TE insertions, 1494 were within the genic regions of 551 genes (see Supplementary Table S4). Out of 151 somatic TE insertions, 148 tumor-specific insertions were associated with a total of 79 genes (see Supplementary Table S4), while 3 genes DLC1, UBE2F, and UBE2F-SCLY (see Supplementary Table S4) were affected by the normal-specific insertions (see Fig. 3c). Since these categories of TE insertions (i.e., germline TE insertions, tumor-specific somatic TE insertions, and normal-specific somatic TE insertions) were determined based on the individual patient, we observed overlaps among the corresponding gene sets (see Fig. 3c). Functional enrichment analysis of the 551 genes affected by germline TE insertions and 65 genes exclusively affected by tumor-specific somatic TE insertions were explored as well. As shown in Fig. 3d, genes disrupted by germline TE insertions are enriched in the neuronal synapse, cell junctions, also related to various transcription factors via the unique motifs the gene contains, whereas genes exclusively associated with tumor-specific somatic TE insertions are involved in the enriched pathways in cancer development (see Fig. 3e).

**Figure 4:**
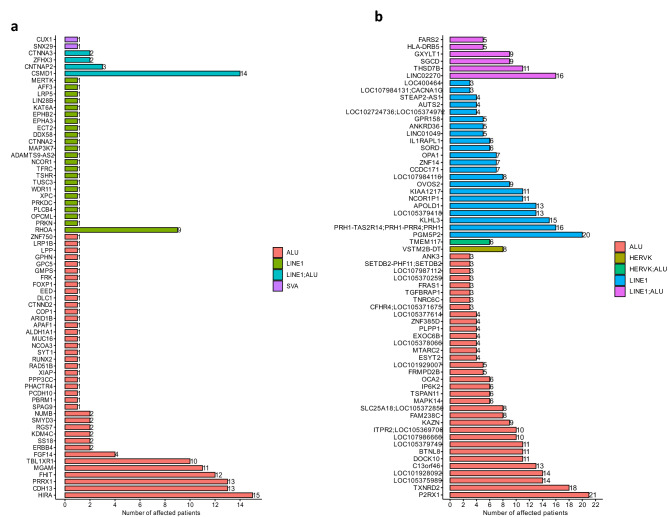

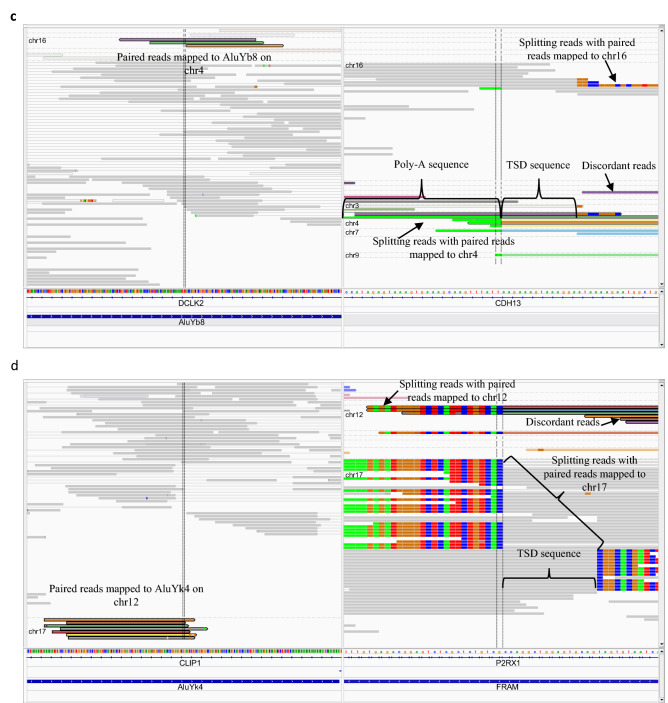
Genes that are affected by OS patient-specific TE insertions. (**a**) The number of OS patients in which cancer-related genes were affected by OS patient-specific TE insertions. (**b**) The number of OS patients in which non-cancer genes were recurrently affected by germline TE insertions (i.e., the same gene affected by insertions in at least 3 patients). (**c**) An example of germline TE insertion in the intron of CDH13 gene (located on chromosome 16) where 3 paired reads associated with splitting reads were completely mapped to the AluYb8 subfamily on chromosome 4 (TSD: AAGAAAGTAAAGGA). (**d**) An example of germline TE insertion in the intron of P2RX1 gene (located on chromosome 17) where 7 paired reads associated with splitting and discordant reads were mapped to the AluYk4 subfamily on chromosome 12 (TSD: CCAGGCTGGAGTGCAG).

To explore the association between OS patient-specific TE insertions identified in this study and cancer-associated genes, 2682 cancer-associated genes were retrieved from public databases (i.e., COSMIC Cancer Gene Census^28^, TSGene^29^, IntOgen^30^, oncogene database^31^, and OncoKB Cancer Gene List^32^, see Supplementary Table S5) and their chromosomal coordinates were compared to our TE insertion sites. Particularly, 68 cancer-associated genes (including 32 oncogenes (OG) and 36 tumor suppressor genes (TS), see Supplementary Table S6) were affected by OS patient-specific TE insertions (see Fig. 4a). Among these 68 genes, 15 were affected by tumor-specific somatic TE insertions, while 52 were exclusively disrupted by germline TE insertions, and only one (DLC1) was affected by normal-specific somatic TE insertions as detailed in Supplementary Table S6. The top 10 frequently affected cancer-associated genes are HIRA (OG), CSMD1 (TS), CDH13 (TS), PRRX1 (OG), FHIT (TS), MGAM (OG), TBL1XR1 (OG), RHOA (OG), FGF14 (TS), and CNTNAP2 (TS). At least 3 out of 39 individual patients were affected by TE insertions in these genes. Among these 68 cancer genes, 64 were affected by TE insertions in their intronic regions, and the remaining 4 were either in their 5′UTR, 3′UTR, or CDS regions (see Supplementary Table S6). Among 64 intronic TE insertions, 3 insertions were within 100 bp from the nearest exons (see Supplementary Table S6). To further verify TE insertions detected in this study by the consensus approach as true cases, alignments of the reads were visualized and examined carefully in the IGV as described in the method section. The paired-end reads (namely, the read pairs of two mate reads) sampled from TE insertion sites can come with different configurations, some of which could help manually validate TE insertions detected by different tools. As shown in Supplementary Fig. S7, paired-end reads can contain splitting reads where a portion of one mate read can be mapped to the TE reference while its left portion can be properly mapped to the reference genome, and the splitting junction can be used to identify the TE insertion site in the genome precisely. Dependent on where paired-end reads were sampled during sequencing, one mate read can be entirely mapped to either the TE reference or the reference genome. The paired-end reads can also be discordant reads where one mate read is properly mapped to the reference genome sequence entirely while the other mate is mapped to the TE reference entirely. CDH13 proved to be a tumor suppressor gene in a variety of cancers^33^. An IGV visualization example of one germline Alu insertion in the intron of CDH13 gene (located on chromosome 16) from one of the OS patient tumor samples was shown in Fig. 4c. The right part of the panel shows splitting reads and discordant reads that support the Alu insertion in the intron of CDH13 based on the repeat masker annotation track in IGV. Splitting reads containing soft-clipped mismatches are colored based on different mismatches. Discordant reads are highlighted with different colors representing chromosomes from which paired mates are mapped. Specifically, two different types of splitting reads were shown here as the evidence to support the TE insertion. In the first type, paired mates for 3 splitting reads (see the arrow on the top right part of the right panel) were mapped to the reference genome (i.e., chromosome 16 in this case). For the second type, paired mates for 3 splitting reads (see the arrow in the lower left part of the right panel) were completely mapped to AluYb8 subfamily on chromosome 4 as indicated on the top left panel. In addition to chromosome 4, as indicated in the lower left part of the right panel, paired mates for other splitting reads can also be mapped to either chromosome 3, 7 and 9. Based on the number of paired mates that can be mapped to different type of TEs on different chromosomes, the TE subfamily with the largest number of mapped paired mates was used to annotate a given TE insertion. Some LINE-1 mediated TE insertions also contain poly-A sequence as evident in the right panel (i.e., bases colored in green). Based on splitting reads, TSD sequence can also be identified as indicated in the right panel, which is AAGAAAGTAAAGGA and the two vertical lines in the right panel indicated the insertion site on the reference genome.

Besides cancer-associated genes, genes that were recurrently affected by OS patient-specific TE insertions may also be important and putative biomarkers in the progression of the disease. By using a threshold of 3 (i.e., at least 3 patients with the same gene affected by OS patient-specific TE insertions^16^), a total of 81 recurrently affected genes were exclusively identified in germline TE insertions, which includes 9 known cancer genes, as detailed in Supplementary Table S7. The top 10 recurrently affected non-cancer genes include P2RX1, PGM5P2, TXNRD2, LINC02270, PRH1, KLHL3, LOC105375989, LOC101928092, LOC105379418, and C13orf46 as shown in Fig. 4b, where each of these genes was affected in at least 13 out of 39 OS patients. Among these non-cancer genes, germline TE insertions in the P2RX1 gene (located on chromosome 17) affected 21 OS patients. One visualization example of this insertion via IGV was shown in Fig. 4d, where 7 paired mates (see the lower part of the left panel) corresponding to 3 splitting and 4 discordant reads (see arrows on top of the right panel, one discordant read was not shown) were completely mapped to the AluYk4 subfamily located on chromosome 12. In this case, the poly-A sequence was not inserted in the genome and the TSD sequence associated with this AluYk4 insertion is CCAGGCTGGAGTGCAG. Similarly, the two vertical lines in the right panel indicated the insertion site on the reference genome.

### Dysregulation of DNA methylation in OS tumors

Given up-regulated TE expressions observed in OS tumor samples, we then analyzed the DNA methylation data of OS tumor samples and compared them to DNA methylation levels of normal osteoblast cell lines. We found that except gene body regions, overall methylation levels in the rest of the genomic regions (i.e., TSS1500, TSS200, 5′UTR, 1stExon, and 3′UTR) were relatively higher in OS tumor samples compared to those of normal osteoblast cell lines as shown in Supplementary Fig. S8a.

**Figure 5:**
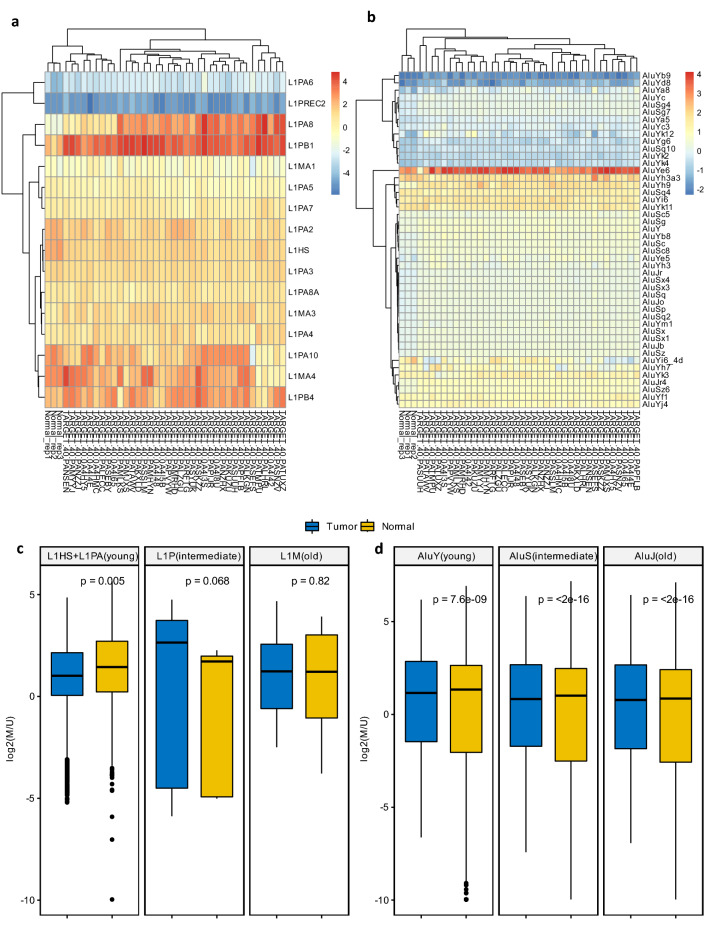
The comparison of methylation levels associated with Alu and full-length LINE-1 between OS tumor samples and normal controls. (**a**) The full-length LINE-1 methylation levels (namely, averaged mValue of probes corresponding to +/− 500 bps flanking the most 5′ end of LINE-1 for each subfamily of full-length LINE-1) across OS tumor samples and normal controls. (**b**) The Alu methylation levels (averaged mValue of probes corresponding to +/− 500 bps flanking the most 5′ end for each subfamily of Alu) across OS tumor samples and normal controls. (**c**) The comparison of methylation levels for full-length LINE-1 of different evolutionary ages between OS tumor samples and normal controls. (**d**) The comparison of methylation levels for Alu of different evolutionary ages between OS tumor samples and normal controls. (Non-parametric unpaired two-sample Wilcoxon tests were used for the statistical test between methylation levels associated with TEs (e.g., Alu and full-length LINE-1) in OS tumor samples compared with normal controls.).

To test if the methylation profile of TEs follows the trend observed genome-wide, the genomic coordinates that correspond to the 1 kb transcription start site (TSS) region (namely, +/− 500 bps flanking the most 5′ ends) of Alu and full-length LINE-1 in hg38 repeat regions were extracted and the relevant methylation levels were compared between OS tumor samples and normal controls. The hg38 repeat regions contain 1,131,306 annotated Alu elements that belong to 46 Alu subfamilies and 961,456 annotated LINE-1 elements corresponding to 127 LINE-1 subfamilies. The length distributions of all annotated LINE-1 and Alu elements were shown in Supplementary Fig. S8b,c, respectively. The majority of LINE-1 elements were truncated in the human genome (namely, less than 6 kb). Of all annotated LINE-1, a total of 7413 full-length LINE-1 were identified. The intersections between 1 kb TSS regions of these identified Alu, as well as full-length LINE-1, and genomic coordinates of the 450 K DNA methylation probes were obtained. Out of 427,954 probes, 39 were excluded from further analysis due to the failure of their coordinate conversions from hg19 to hg38. With the remaining 427,915 probes, a total of 36,841 probes were within 1 kb TSS regions of either Alu (36,771 mapped to Alu) or full-length LINE-1 (70 mapped to LINE-1). Among 36,771 probes mapped to 1 kb TSS regions of Alu, 4625 were identified within multiple Alu subfamilies and excluded from further analysis to reduce ambiguity. As indicated in Supplementary Fig. S8d,e, among 70 probes uniquely mapped to 1 kb TSS regions of full-length LINE-1, 16 LINE-1 subfamilies were identified, and among 32,146 probes uniquely mapped to 1 kb TSS regions of Alu, 46 Alu subfamilies were identified. The methylation levels (mValues: log2(Beta value/(1 − Beta value))) associated with these Alu and full-length LIINE-1 were then averaged within each TE subfamily for each OS tumor sample and normal control, respectively. The comparison of methylation levels between OS tumor samples and normal osteoblast cell lines were represented by heatmaps as shown in Fig. 5a,b, for 16 subfamilies of full-length LINE-1 and 46 subfamilies of Alu, respectively. It is noticeable that methylation levels of both full-length LINE-1 subfamilies and Alu subfamilies can be used to distinguish normal osteoblast cell lines from OS tumor samples as indicated by their distinct clusters. To further compare the methylation levels associated with Alu and full-length LINE-1 between OS tumor samples and normal osteoblast cell lines based on evolutionary ages of TEs, 16 subfamilies of LINE-1 were categorized into L1HS + L1PA (young), L1P (intermediate), and L1M (old) while 46 Alu subfamilies were categorized into AluY (young), AluS (intermediate) and Alu (old) as described previously^34^. Unlike the hypermethylation observed genome-wide in OS tumors, the methylation level of L1HS + L1PA (young) was significantly lower in tumor samples than normal osteoblast cell lines; the methylation levels of all Alu categories were also significantly lower in tumor samples than in normal osteoblast cell lines albeit to a lesser degree, as shown in Fig. 5c,d, respectively.

### Correlations among methylation, expression, insertion of TE in OS tumor samples, and patient event-free survival

**Figure 6:**
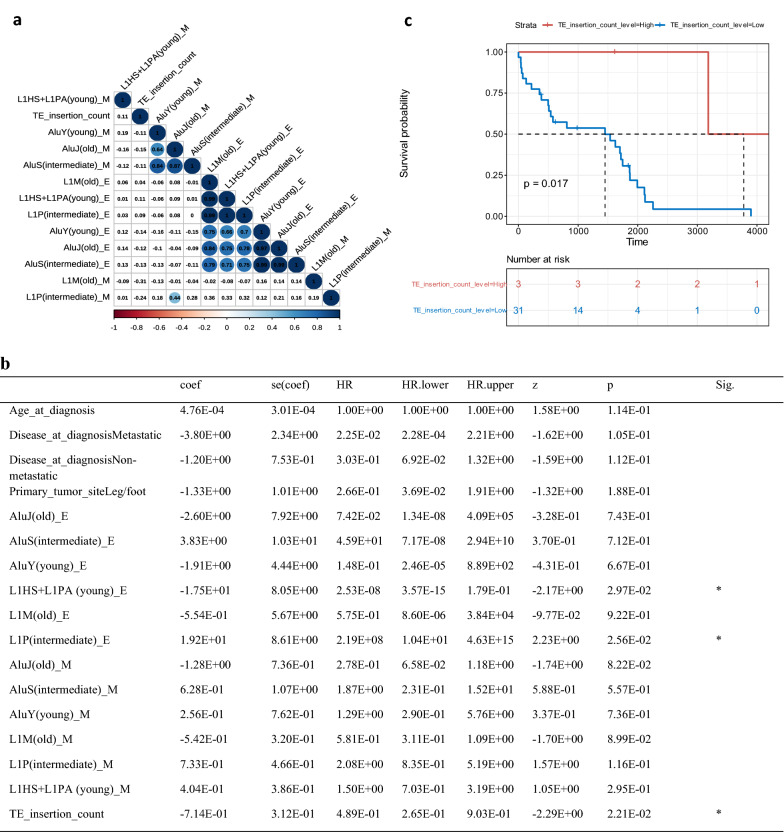
The correlations among TE methylation, expression, insertion, and patient outcome. (**a**) The correlations among levels of TE (including LINE-1, Alu of different evolutionary ages) expression, methylation, and TE (including LINE-1, Alu, SVA, and HERV-K) insertion frequencies in OS tumor samples. (TE ends with E represents expression levels whereas TE ends with M represents methylation levels; the color bar at the bottom indicates correlation levels with red representing the negative correlation and blue representing the positive correlation; the non-parametric Spearman rank correlation test was used for the correlation analysis with significant correlations (*p* < 0.05) color-filled). (**b**) The results of the Cox proportional hazards model with standardized TE activities [i.e., expression level, the methylation level of TE (including LINE-1 and Alu of different evolutionary ages) and insertion frequencies of TE (including LINE-1, Alu, SVA, and HERV-K)] in OS tumor samples and available clinical data (age at diagnosis, disease at diagnosis, and primary tumor site) as predictors for the patient event-free survival (*p* value was calculated based on the likelihood ratio test, predictors that are significantly associated with the event-free survival are indicated in the last column by the * (*p* < 0.05). (**c**) The probability of the patient event-free survival stratified by patients with higher TE insertions in their tumor samples (more than 100 TE insertions) and patients with a lower number of TE insertions in their tumor samples (less than 100 TE insertions) depicted by Kaplan–Meier curve (Log-rank test was used to calculate the *p* value). HR: Hazard ratio, z: Z-score, Likelihood ratio test = 28.12 on 17 df, *p* = 0.0436, n = 34, number of events = 29.

The expression levels of TEs can be in part regulated by their methylation statuses and can potentially affect TE insertion frequencies in the genome^10^. To explore associations among TE methylation, expression, and insertions, the methylation, expression, and insertion data of TEs in OS tumor samples were analyzed. As shown in Fig. 6a, methylation levels of Alu of different evolutionary ages show a significantly positive correlation to each other (e.g., the higher/lower the methylation level of AluY(young), the higher/lower the methylation level of AluS(intermediate) or AluJ(old)), which is also reflected by the positive correlation for their expression levels (e.g., the higher/lower the expression level of AluY(young), the higher/lower the expression level of AluS(intermediate) or AluJ(old)). Even though methylation levels of LINE-1 of different evolutionary ages did not show a significant correlation to each other, their expression levels on the other hand are positively correlated (i.e., the higher/lower the expression level of LINE-1 (young), the higher/lower the expression level of LINE-1 (intermediate) or LINE-1 (old)). Furthermore, a positive correlation was also observed between expression levels of Alu and LINE-1. We also noticed that TE (including LINE-1, Alu, SVA, and HERV-K) insertion frequencies in OS tumor samples were negatively correlated with methylation levels of L1P (intermediate), L1M (old), AluY (young), AluS (intermediate), and AluJ (old), but positively correlated with expression levels of L1HS + L1PA (young), L1P (intermediate), and L1M (old), however, the correlations were not statistically significant. Finally, we failed to observe any significant correlations between expression and methylation levels of TEs in OS tumor samples.

To explore potential clinical applications of TE activities for OS patients, the Cox proportional hazards model was used to investigate the association between activities of TEs in OS tumor samples and the patient event-free survival time. The number of TE insertions (including LINE-1, Alu, SVA, and HERV-K) and expression levels of both evolutionarily young and intermediate LINE-1 in OS tumor samples were significantly associated with the patient’s event-free survival time (*p* < 0.05), as indicated in Fig. 6b. In particular, a higher level of TE insertions and expression of evolutionarily young LINE-1 is associated with a longer time of event-free survival, whereas the higher expression level of evolutionarily intermediate LINE-1 is associated with a shorter time of event-free survival. Among 34 patients analyzed for their event-free survivals, 3 patients had more than 100 TE insertions in their tumor samples and the Kaplan–Meier curve shown in Fig. 6c indicated that, compared to patients with lower TE insertions (less than 100), patients with a higher number of TE insertions in their tumor samples had a higher probability of event-free survival, which is consistent with the result from Fig. 6b.

## Discussion

While investigations of the importance of TE insertions in the development and prognosis of various cancers have been previously explored^14,16,19,35^, the alteration of TE expression, insertional landscape, dysregulation of related methylation status as well as their clinical implications have not been systematically analyzed yet in OS patients.

Due to the lack of matched RNA-seq data of normal tissue samples for OS patients from the TARGET phs000468 study, to compare the expression level of genes and TEs between OS tumor samples and normal controls, additional paired-end RNA-seq data were incorporated from a recent study (SRP193919)^20^. The correlation among all samples in terms of their gene expression profiles at the gene transcript level indicates the similarity between the OS tumor samples from both the TARGET phs000468 study and SRP193919 study (see Supplementary Fig. S1a). By using TEtranscripts^36^, which demonstrated superior performance for TE expression measurement followed by DESeq2^37^, we showed that compared with samples of normal muscle tissues adjacent to osteosarcoma, LINE-1 and Alu of different evolutionary ages, several subfamilies of SVA and HERV-K and various satellite repeats were all significantly up-regulated in OS tumor samples. This result is consistent with observations that expression levels of LINE-1 in breast invasive carcinoma, head and neck squamous cell carcinoma, and lung adenocarcinoma were all up-regulated^17^ and over-expressions of various satellites were found in human and mouse carcinomas^13,38^. During TE insertions, double-stranded DNA breakages can be triggered, which would lead to activated DNA repair responses^13,39^. By comparing expression levels of genes involved in homologous recombination, non-homologous end-joining, and mismatch repair processes between OS tumor samples and samples of normal muscle tissues adjacent to osteosarcoma, we found that many of these genes were up-regulated in OS tumor samples, which could be either due to the enhanced TE insertion activities in OS tumors, or the increased level of DNA damages associated with tumor progression or both. Due to the limitation of available RNA-seq data, the normal control samples used in this study, as well as in a recent study^20^, were paired-normal muscle tissues adjacent to osteosarcoma. Therefore, the interpretation of the expression profile differences between bone tissue where OS is derived and adjacent normal muscle tissues need to be cautious due to the tissue differences. Ideally, bone tissues should be collected as the normal control in future studies when focusing on the differential gene expression analysis between OS and normal controls. Nevertheless, based on the upregulation of TE expressions in many other cancers, we expect to see similar results in OS tumors.

Given the difficulty in TE insertion identification based on short reads generated from NGS platforms^40^, a consensus approach to reduce false positives was taken in this study for TE insertion detections combined with human validation of alignment visualization (see Fig. 4c,d). Even though the total number of TE insertions identified in OS tumors was higher than that in the paired normal samples (22,001 in tumors vs 17,598 in normal samples), it should be noted that the lower sequencing depths in normal samples (see Supplementary Fig. S2a) could potentially reduce the number of detectable TE insertions. To characterize TE insertional features and identify TE insertions that are specific in OS patients, LINE-1 mediated TE insertions reported in 1KGP were used as a reference to detect both somatic and germline insertions existing only in OS patients. One of such insertional features important for accurate detection of novel LINE-1 mediated TE insertions is the TSD^40^. We found that the length distributions of TSDs associated with LINE-1 mediated TE insertions detected in OS patients and reported in 1KGP were both centered around 15 bps (see Fig. 2a,b), which was consistent with other studies^19,41^. Similarly, we found that LINE-1 mediated TE insertions preferentially occurred in AT-rich regions of the genome as previously reported^41^, whereas no discernable pattern of nucleotide base compositions in terms of the preference of HERV-K insertions was identified in OS patients (see Supplementary Fig. S3), presumably due to the different insertion mechanism for HERV-K insertions compared to LINE-1 mediated TE insertions^41^. As for the genomic context of LINE-1 mediated TE insertions, consistent with previous studies^15–17^, insertions identified in OS patients and reported in 1KGP tend to occur in either intergenic or intronic regions (see Fig. 2e,f), which were suggested to be associated with passenger mutations in terms of tumor development^10^. Similar to a previous study^19^, we also found that TE insertions tend to target longer genes. CFS has been associated with TE insertions in various cancers^16,19^. We found that most CFS (114 out of 124 in OS patients and 116 out of 124 in 1KGP) were associated with LINE-1 mediated TE insertions. In 1KGP, more than 2000 individuals were analyzed by MELT^21^ for the identification of LINE-1 mediated TE insertions^25^. In this study, with only 39 OS patients and using a consensus approach (MELT^21^ and Mobster^22^), we identified more LINE-1 mediated TE insertions (39,574) than that reported in 1KGP (16,676), indicating elevated insertions in OS patients. Nonetheless, only 2.68% of annotated genes were affected by insertions in OS patients compared to 6.75% in 1KGP, which could be due to the observation that LINE-1 mediated TE insertions in OS patients were enriched in some regions of the genome compared to a more spread-out nature of TE insertions observed in 1KGP (see Supplementary Fig. S4). This concentrated insertion preference along the OS patient genomes could be potentially important for OS development, which is worthy of further investigations.

Increasing amounts of evidence suggested that activated human TEs not only can contribute to the genomic instability that is widely observed in cancer patients but also can be causatively linked to the development of cancers^10,16^. It is therefore important to distinguish polymorphic TE insertions from disease-specific TE insertions. With different distance thresholds, we found that a window size of 20 bps can effectively eliminate most of potentially polymorphic TE insertions in OS patients, resulting in a total of 3326 OS patient-specific TE insertions identified, which were further validated by manual visualizations using alignments, as evident in Fig. 4c,d. We also observed the extensive heterogeneity among patients in terms of TE insertion frequencies, which is consistent with the observation in a recent study^19^. It has been shown that both inherited predispositions through germline mutations and acquired somatic mutations can contribute to the development of cancers^42^, in which the genomic context (e.g., susceptibility to a certain disease) established by germline mutations can often influence the biological impacts of somatic mutations^43^. Therefore, to better understand the occurrence of cancer, the study of both somatic and germline mutations is necessary. Compared with other studies^16,17^ focusing primarily on somatic TE insertion detections using paired tumor-normal samples, we also examined and visually validated germline TE insertions among the identified OS patient-specific TE insertions. Consistent with the observation that somatic TE insertions tend to occur in cancers of epithelial origins^14^, we found that most OS (a type of cancer derived from mesenchyme) patient-specific TE insertions were germline insertions (3175 out of 3326), with few somatic insertions (151 out of 3326). A recent pan-cancer study focusing on TE insertions (i.e., LINE-1, Alu, SVA, and HERV-K) with a different insertion detection tool also found that among all osteosarcoma patients (n = 47) analyzed, only about 10% of the samples contain somatic insertions ranging from 1 to 10 per sample (patient), which is significantly depleted in comparison with other types of cancers (e.g., esophageal adenocarcinoma, head-and-neck squamous carcinoma, lung squamous carcinoma, and colorectal adenocarcinoma)^44^. This observation agrees with previous studies for the lack of somatic TE insertions in cancer of non-epithelial origins^14,15^. Furthermore, about 80% of all LINE-1 mediated TE insertions identified in breast invasive carcinoma, head and neck squamous cell carcinoma, and lung adenocarcinoma were also determined as germline insertions^17^. All these results support the validity of the reasonably small number of somatic TE insertions identified in this study. Nonetheless in this study, we found a total of 148 tumor-specific somatic TE insertions among 39 OS patients, and 27 of these patients (69%) had such insertions ranging from 1 to 23 while the rest of 12 patients had 0 insertions (see Fig. 3b).

The functions of affected genes associated with germline TE insertions were enriched in the neuronal synapses as well as cell junctions, which is consistent with the identified biological functions associated with somatic TE insertions in a recent study^19^. Furthermore, many transcription factors (TF) associated with cancer development were also found to be enriched in the genes affected by germline TE insertions through our enrichment analysis (due to the genes that can be recognized by TFs were enriched in germline TE affected genes). For example, genes containing TAATHA motif that can be recognized by the TF PMX1 were enriched in genes affected by germline TE insertions. PMX1 has been associated with the epithelial-mesenchymal transition in various cancer studies^45,46^. Similarly, TF Sox-6 can recognize genes containing the CACCGAACAAT motif that is also enriched in the genes affected by germline TE insertions. In the esophageal squamous cell carcinoma, Sox-6 was reported as a tumor suppressor gene^47^ whereas Sox-6 was shown to possess a strong oncogenic property in Ewing sarcoma^48^. In addition, other cancer-related TFs were also found in our enrichment analysis, including ALX3 (in neuroblastoma)^49^, HOXA6 (in clear renal cell carcinoma)^50^, HOXC-8, FOXO1 (in breast cancer)^51,52^, PARP (in neuroblastoma, endometrial cancer, breast cancer, and malignant lymphoma)^53–56^, IPF1 (in pancreatic neuroendocrine neoplasms)^57^, and HOXB2 (in glioma)^58^. Therefore, by focusing on germline TE insertions following identification of OS patient-specific TE insertions, we revealed the typically overlooked importance of germline TE insertions in the development of OS. Given its earlier onset, OS has been frequently associated with germline predisposition syndromes^5^. Our analysis also suggests that germline TE insertions in OS patients can disrupt similar biological functions that are important in cancer development as affected by somatic TE insertions in other types of cancers. Considering that OS typically affects young adults^1^ and the occurrence of cancer is generally explained by the accumulation of mutations that are important for cell proliferation, it is reasonable to speculate that these germline TE insertions observed in OS patients might comprise part of hereditary factors that can predispose patients to OS and can similarly contribute to the tumorigenesis as somatic TE insertions do in other types of cancers. In fact, by correlation analysis between the germline variant burden, somatic mutation burden, and age of diagnosis for cancer patients in three databases (namely, The Cancer Genome Atlas (TCGA), Pan-cancer Analysis of Whole Genomes (PCAWG), and the UK Biobank (UKBB)), a recent study showed that patients who developed cancer at a younger age had a larger number of high-functional-impact germline variants (gHFI) in cancer hallmark genes than do patient who developed cancer at an older age. Furthermore, patients who developed cancer at an older age were more reliant on acquired somatic mutations in the cancer genes, and there often exists a negative correlation between the average number of gHFI and somatic mutation in older patients^59^. These observations support the notion of functional complementarity of germline mutations and somatic mutations in the development of cancer and suggest that as a type of important germline mutations, germline TE insertions identified in this study might serve as an important piece of information to understand the development of OS. However, the relationship between germline mutations and somatic mutations in the development of cancer in general needs further exploration. In terms of the genes that are exclusively affected by tumor-specific somatic TE insertions, we also observed the enrichment of affected genes in the cancer pathway (due to tumor-specific TE insertions in genes such as COL4A6, PLCB4, ARNT2, LRP5, NCOA3, and CTNNA2).

It has been reported that TE insertions in cancer-associated genes can potentially be causative in cancer development^16^. In this study, a total of 68 cancer-associated genes were found to be affected by OS patient-specific TE insertions, where most of the insertions (64 out of 68) occurred in intronic regions, which may trigger epigenetic modifications (e.g., methylation)^60^ thus affecting the transcriptional activities of nearby genes. In addition, 3 out of 64 insertions were found within 100 bps from the nearest exons, which may affect the splicing process and generate aberrant transcript isoforms. In addition to cancer genes, we also observed some non-cancer genes recurrently affected by germline TE insertions in OS patients. Specifically, a total of 72 recurrently affected non-cancer genes were identified in at least 3 OS patients. Among the top 10 (affecting more than 13 out of 39 OS patients in this study) recurrently affected non-cancer genes, germline TE insertions of P2RX1 affected 21/39 OS patients and was recently shown to be associated with the immunosuppressive microenvironment in pancreatic ductal adenocarcinoma liver metastases^61^. PGM5P2 is a pseudogene that was affected by germline TE insertions in 20 out of 39 OS patients. A recent study on the chordoma (a rare bone tumor with unknown etiology and high recurrence rate) identified the deletion of PGM5P2 among others in the 9p21.11 deletion peak, although its associated biological functions with chordoma are still unclear^62^. Furthermore, a study focusing on the diagnostic value of differentially expressed genes (DEGs) in formalin-fixed, paraffin-embedded prostate tumor samples identified that PGM5P2 as one of the top 5 DEGs that were down-regulated in prostate tumor samples^63^, indicating the potential tumor-suppressive nature of PGM5P2. Furthermore, we found that genes including LINC02270, PRH1, and KLHL3 were affected by germline TE insertions in 16, 16, and 15 OS patients, respectively, although their cancer-associated functions have never been reported before. Similarly, uncharacterized genes including LOC105375989, LOC101928092, LOC105379418, C13orf46 were affected by germline TE insertions in 14, 14, 13, and 13 OS patients, respectively. Although the underlying molecular connection between disruptions of these non-cancer genes and OS development has been elusive, given the high frequency of TE insertions in these genes, they could be potentially served as OS-specific oncogenes or tumor suppressor genes and their roles in the OS development is worthy of further investigations.

The hypermethylation of individual tumor suppressor genes coupled with global hypomethylation in a larger genomic context is frequently observed in different types of cancers^18^. Therefore, it has been suggested that regulations for TE activities are typically compromised due to the genome-wide hypomethylation, which could be conducive for more TE expression and consequently more frequent TE insertions^10^. Unlike the global hypomethylation reported in other cancers, in this study, we found that overall methylation levels observed in OS tumor samples were relatively higher compared to that of normal osteoblast cell lines. However, the interpretation of our data analysis result requires some scrutiny. This is because of the following reasons: (1) normal controls (osteoblast cell lines) used for the comparison of methylation levels are not directly from tumor-paired normal samples; (2) unlike the whole-genome bisulfite sequencing which can measure the methylation status genome-wide with a single-base resolution, 450 K methylation array preferentially focuses on CpG island and gene promoter regions^64^ as evident by the fact that only 32,146 out of 1,131,306 Alu elements and 70 out of 7413 (or 16 out of 127 subfamilies) full-length LINE-1 elements were successfully covered by 450 K probes. Therefore, to fully understand the methylation profile of OS tumors, further studies are needed. Nonetheless, since a large proportion of human genomes are TEs and DNA methylation is also one of the important ways for hosts to regulate the TE movement, given the global hypomethylation found in various cancers it is reasonable to speculate that the global hypomethylation observed in cancer genomes might be attributed to the demethylation of these repeated DNA sequences^65^. In fact, hypomethylation of LINE-1 has been discovered in a variety of cancers including colorectal cancer, gastric cancer, prostate cancer, head-and-neck cancer, etc.^65^. Consequently, the overexpression of LINE-1 has been reported in various human cancers^17^. Consistent with these observations, we found that methylation levels of L1HS + L1PA (young) and Alu of different evolutionary ages were relatively lower in OS tumor samples compared to normal osteoblast cell lines. Furthermore, we noticed that methylation profiles of LINE-1 and Alu of different subfamilies can be used to differentiate OS tumor samples from normal osteoblast cell lines, suggesting their potential applications for early OS detection.

The methylation status of TEs supposedly plays an important role in the TE expression regulation and thus affects TE insertion frequency. Given the lack of methylation data for tumor-paired OS normal controls, by restricting our analysis to OS tumor samples, we showed a significantly positive correlation among methylation and expression levels of Alu of different evolutionary ages. Similarly, expression levels of LINE-1 of different evolutionary ages were positively correlated. However, we failed to observe any significant correlations between methylation and expression levels for Alu and LINE-1 in OS tumor samples, which might be because the 450 K methylation array cannot offer a more comprehensive measurement of methylation status associated with Alu and LINE-1, therefore affecting our correlation analysis. The positive correlations observed in OS tumor samples for the expression levels of LINE-1 and Alu were probably because they were all up-regulated in OS tumor samples. Regarding the potential clinical application of TE activities for cancer patients, a recent study focusing on TE insertions in colorectal cancer showed that a higher number of somatic TE insertions was associated with poorer disease-specific survival^16^. In OS patients, by integrative analysis of TE activities, we found that a higher level of TE insertions (including both germline and somatic TE insertions) and expression of evolutionarily young LINE-1 in tumor samples are associated with a longer time of the patient event-free survival. With the Kaplan–Meier curve, we further showed that patients with more than 100 TE insertions in their tumor samples achieved a higher probability of event-free survival compared to patients with a lower number of TE insertions. However, the prognostic values of TE insertions for OS patient event-free survival obtained in this study need to be validated in a bigger sample-sized data in future studies and its underlying mechanisms also need to be explored.

## Methods

### RNA-Seq data analysis

Out of 39 OS patients with WGS data, 36 have paired-end RNA-Seq data of tumor samples, which were retrieved from the TARGET phs000468 study via the SRA-toolkit (https://trace.ncbi.nlm.nih.gov/Traces/sra/sra.cgi?view=software). To compare the expression of genes and TEs between tumor samples and normal controls, additional paired-end RNA-Seq data containing 4 samples of normal muscle tissue adjacent to osteosarcoma and 16 OS tumor tissue samples were incorporated from a recent study (SRP193919)^20^. For SRA data, the parallel-fastq-dump (https://github.com/rvalieris/parallel-fastq-dump) was used to extract paired reads. The workflow for the RNA-Seq analysis was depicted in Supplementary Fig. S9a and detailed in supplementary methods (see the section “RNA-seq data analysis” on page 2).

All LINE-1 subfamilies identified in RNA-seq results were grouped into three LINE-1 categories: L1HS + L1PA (young), L1P (intermediate), and L1M (old), and all subfamilies of Alu were grouped into three Alu categories: AluY (young), AluS (intermediate), and Alu (old), according to their evolutionary ages^34^. The expression levels of all categories of LINE-1 and Alu and subfamilies of SVA and HERV-K in OS tumor samples and samples of normal muscle tissue adjacent to osteosarcoma as control were compared. Because TE insertions could cause double-stranded DNA breaks, which will induce DNA damage responses from the host, 26 genes involved in the homologous recombination, 11 genes involved in the non-homologous end-joining, and 14 genes involved in DNA mismatch repairs were obtained from a recent study^66^ (see Sub-Table2) and their expression levels in tumor samples and normal controls were also compared. To obtain expression levels of individual genes, the normalized expression data of all transcript isoforms corresponding to a given gene were averaged and compared between OS tumor and normal controls.

### WGS data analysis

WGS data from tumor tissue and paired blood-derived normal samples of 39 OS patients were downloaded from the TARGET phs000468 study via SRA Toolkit (version: 2.10.0). The data quality control followed a similar strategy as mentioned in the RNA-Seq data analysis. The workflow for TE insertion detection using WGS data was depicted in Supplementary Fig. S9b. Specifically, the bwa mem aligner (version: 0.7.17)^67^ was used to align clean reads to the hg38 reference genome with default parameters. The generated sequence alignment map (SAM) files were subsequently converted to the binary alignment map (BAM) files and sorted, indexed with samtools^68^. MELT^21^and Mobster^22^ were used for TE insertion detections using WGS data due to their demonstrated performance in recent benchmarking comparisons^23,24^, both of which take BAM files as inputs. Given the difficulty in the identification of TE insertions based on short reads generated from the NGS platform^40^, consensus TE insertion sites detected from both tools were used for subsequent analysis. Specifically, predictions of TE insertions in each genome (sample) made by two tools were considered to represent the same insertions if they were found within 100 bp of each other^17^. Mobster reports imprecise TE insertion sites and associated confidence intervals around insertion sites^22^ whereas MELT reports both TE insertion sites and associated supporting evidence^21^. Accordingly, the insertion position reported by MELT was used as the final insertion site of each consensus TE insertion for subsequent data analysis.

To validate characteristics of LINE-1 mediated TE insertions identified in OS patients, LINE-1 mediated TE insertions reported in 1KGP study using the human genome h19 reference^25^ (https://ftp-trace.ncbi.nih.gov/1000genomes/ftp/release/20130502/) were downloaded and used for comparison as detailed in supplementary methods (see the section “Validation for characteristics of LINE-1 mediated TE insertions” on page 2). Among TE insertions identified in this study, the ones that are specific for OS patients (namely, OS patient-specific TE insertions) are presumably important in OS development. To identify OS patient-specific TE insertions, genomic coordinates of LINE-1 mediated TE insertions reported in 1KGP were used to eliminate potentially polymorphic LINE-1 mediated TE insertions among TE insertions identified in OS patients (HERV-K was not filtered due to the lack of HERV-K identification in 1KGP) as detailed in supplementary methods (see the section “Identifications of OS patient-specific TE insertions” on page 3). Furthermore, OS patient-specific TE insertions were grouped into germline TE insertions and somatic TE insertions for each patient, by using a window size of 100 bps^17^ followed by IGV validation as detailed in supplementary methods (see the section “Identifications of germline and somatic TE insertions” on page 3). The somatic TE insertions include both normal-specific somatic TE insertions and tumor-specific somatic TE insertions.

To explore associations of OS patient-specific TE insertions with established cancer genes, a total of 2682 cancer-related genes from COSMIC Cancer Gene Census^28^, TSGene^29^, IntOgen^30^, oncogene database^31^, and OncoKB Cancer Gene List^32^ were retrieved and analyzed. To further pin down potentially important non-cancer genes that were disrupted by OS patient-specific TE insertions, which might be involved in the development of OS, genes that were recurrently affected by TE insertions (i.e., at least 3 patients having the same gene affected by OS patient-specific TE insertions) were also explored. Functional annotation of genes associated with different categories of TE insertions was analyzed by using g: Profiler^69^.

### DNA methylation data analysis

To compare DNA methylation profiles between OS tumor tissue samples and normal tissue controls, DNA methylation raw data (in idat format generated by IlluminaHumanMethylation450k) from tumor samples of 37 OS patients were retrieved from https://target-data.nci.nih.gov/Public/OS/methylation_array/, with associated metadata. The 50 bp probes employed by the 450 K array can measure the methylation status at single-base resolution for over 450,000 CpGs in the human genome^64^. Due to the lack of DNA methylation data from paired normal samples in OS patients, DNA methylation data (i.e., beta value (proportion of methylation signal for a given probe: M/M + U)) of osteoblast cell lines (3 replicates) retrieved from a previous study^70^ were used as normal controls. The workflow for the methylation data analysis was depicted in Supplementary Fig. S9c.

The annotation associated with each probe was obtained via the R annotation package: IlluminHumanMethylation450kann.ilmn12.hg19 (http://www.bioconductor.org/packages/IlluminaHumanMethylation450kanno.ilmn12.hg19/)^71^ and the conversion of the coordinates from hg19 to hg38 was conducted using pyliftover (https://pypi.org/project/pyliftover/). For the comparison of overall methylation levels between OS tumor samples and the normal osteoblast cell lines, average M values (M value = log2(Beta value/(1 − Beta value))) of all OS tumor samples, and average M values of three normal osteoblast cell lines were stratified by genomic regions associated with each probe including the 1st Exon (probes mapped to the first exon of the genes), 3′UTR (probes mapped to 3′UTR), 5′UTR (probes mapped to 5′UTR), Body (probes mapped to gene body), TSS1500 (probes mapped to 1500 bps upstream of TSS), and TSS200 (probes mapped to 200 bps upstream of TSS). For probes that can be identified in multiple genomic regions, TSS1500 was prioritized followed by TSS200, 5′UTR, the 1st Exon, Body, and lastly 3′UTR.

Given most OS patient-specific TE insertions were associated with LINE-1 and Alu, the methylation status associated with LINE-1 and Alu in OS tumor samples were then compared with normal osteoblast cell lines as detailed in supplementary methods (see the section “DNA methylation data analysis” on page 4).

### Association analysis and patient prognosis

To explore effects of TE methylation levels (i.e., methylation of 1 kb TSS (i.e., +/ 500 bp flanking the most 5′ end) of Alu and full-length LINE-1) on TE expressions (i.e., LINE-1 and Alu) and further on TE (including LINE-1, Alu, SVA and HERV-K) insertion frequencies, the methylation data, expression data, as well as insertion number of TEs in OS tumor samples, were integrated. Specifically, the TE expression data and methylation data were categorized into groups with different evolutionary ages as mentioned before, and average values of TE expression and methylation within each evolutionary group were then computed. Combined with the insertion number of TEs in OS tumor samples, the integrated data set thus represents the activity of TEs in the tumor samples. The integrated data set was then standardized (X − mean(X)/std (X)) and correlations among them were calculated and visualized by using R package corrplot (Version: 0.90, https://github.com/taiyun/corrplot)^72^. To test the prognostic value of TE activities for the OS patient event-free survival, the standardized data corresponding to TE activities in OS tumors and the clinical data including patient age at diagnosis, disease status at diagnosis, and primary tumor sites (obtained from https://target-data.nci.nih.gov/Public/OS/clinical/harmonized/) from 34 OS patients with the complete clinical information were utilized as predictive features. The Cox proportional hazards model and Kaplan–Meier curve implemented in the R survival package (Version: 3.2-7, https://cran.r-project.org/web/packages/survival/)^73^ were used to explore the association between TE activities in OS tumors and the patient event-free survival times.

### Statistical analysis

The statistical significance of comparisons for the expression and methylation data between tumor samples and normal controls was determined using the non-parametric unpaired two-sample Wilcoxon test implemented in the R package ggpubr (Version: 0.4.0, https://github.com/kassambara/ggpubr)^74^ and a *p* value less than 0.05 was considered as a statistically significant difference. In the enrichment analysis of the genes affected by OS patient-specific TE insertions (including both germline and somatic insertions), the hypergeometric test implemented in the g: Profiler (https://biit.cs.ut.ee/gprofiler/gost)^69^ was used for the statistical analysis, and only annotated genes were used for the statistical domain scope, the default g: SCS method was used for multiple testing correction, and the adjusted *p* value < 0.05 was used to select statistically significant terms. For the correlation analysis among TE methylation levels, expression levels, and insertion numbers, the non-parametric Spearman rank correlation test was used. In the Cox proportional hazards model, the *p* value was calculated based on the likelihood ratio test, and the log-rank test was used to calculate the *p* value in the Kaplan–Meier curve.

## Supplementary Information

Supplementary Information.

Supplementary Tables.

## Data Availability

All the data that supports results and conclusions for this study are included in this article and supplementary information.
