# Supplementary material for: The insertion and dysregulation of transposable elements in osteosarcoma and their association with patient event-free survival
Source: Sci Rep. 2022 Jan 10;12:377. doi: 10.1038/s41598-021-04208-5 (PMC8748539; doi:10.1038/s41598-021-04208-5)
Supplement: Supplementary file 1 — Supplementary Information. [file 41598_2021_4208_MOESM1_ESM.docx]

**The insertion and dysregulation of transposable elements in osteosarcoma and their association with patient event-free survival**

Chao Wang and Chun Liang*

[wangc90@miamioh.edu](mailto:wangc90@miamioh.edu); [liangc@miamioh.edu](mailto:liangc@miamioh.edu)

Department of Biology, Miami University, Oxford, Ohio 45056

**Supplementary information**

[Supplementary Methods 1](#_Toc83114333)

[RNA-Seq data analysis 1](#_Toc83114334)

[Validation for characteristics of LINE-1 mediated TE insertions 1](#_Toc83114335)

[Identifications of OS patient-specific TE insertions 2](#_Toc83114336)

[Identifications of germline and somatic TE insertions 2](#_Toc83114337)

[DNA methylation data analysis 3](#_Toc83114338)

[Supplementary Figures 4](#_Toc83114339)

[Supplementary Fig. S1. The expression comparisons for genes of interest between OS tumor samples and samples of normal muscle tissue adjacent to osteosarcoma (normal controls) 6](#_Toc83114340)

[Supplementary Fig. S2. The comparison between TE insertions identified in OS patients and TE insertions reported in 1KGP 7](#_Toc83114341)

[Supplementary Fig. S3. The preference for HERV-K insertions 7](#_Toc83114342)

[Supplementary Fig. S4. The connection between LINE-1 mediated TE insertions and CFSs 8](#_Toc83114343)

[Supplementary Fig. S5. The identification of OS patient-specific TE insertions 9](#_Toc83114344)

[Supplementary Fig. S6. The 3,326 OS patient-specific TE insertions after filtering out polymorphic TE insertions reported in 1KGP 10](#_Toc83114345)

[Supplementary Fig. S7. Validation of TE insertions via IGV 11](#_Toc83114346)

[Supplementary Fig. S8. The comparison of methylation levels between OS tumor samples and normal controls 14](#_Toc83114347)

[Supplementary Fig. S9. Analysis workflow for the measurement of TE activities in OS patients 16](#_Toc83114348)

[Supplementary Tables 16](#_Toc83114349)

[Supplementary Table S1: The subfamilies of major TE elements and their classifications 16](#_Toc83114350)

[Supplementary Table S2: The genes that could be potentially regulated in the host genome following TE insertions. The gene list corresponds to Supplementary Fig. S1c – e 16](#_Toc83114351)

[Supplementary Table S3: The number of insertions mapped to overlapping genomic regions 16](#_Toc83114352)

[Supplementary Table S4: The genes affected by OS patient-specific TE insertions 16](#_Toc83114353)

[Supplementary Table S5: The cancer-associated genes downloaded from public databases 16](#_Toc83114354)

[Supplementary Table S6: The 68 cancer-associated genes affected by OS patient-specific TE insertions 16](#_Toc83114355)

[Supplementary Table S7: The recurrently affected genes by germline TE insertions 16](#_Toc83114356)

[Supplementary references 16](#_Toc83114357)

# Supplementary Methods

## RNA-Seq data analysis

The paired-end reads were cleaned with Trim Galore (version: 0.6.4, <https://github.com/FelixKrueger/TrimGalore>) to remove adaptors and low-quality bases at read ends by Cutadapt (version: 2.6)^1^. The quality of clean reads was assessed with Fastqc (version: v0.11.8)^2^ before their alignments to the reference human genome hg38 with STAR (version: 2.7.4a)^3^. The gene expression at transcript isoform level and TE expression were determined via TEtranscripts (version: 2.1.4)^4^ by following the corresponding protocol^5^. Specifically, the TEcount module in TEtranscripts takes alignment results generated from STAR, the gene annotation file in gtf format downloaded from UCSC Table Browser^6^, and a TE annotation gtf file download from <http://labshare.cshl.edu/shares/mhammelllab/www-data/TEtranscripts/TE_GTF/GRCh38_Ensembl_rmsk_TE.gtf.gz> as inputs, and outputs the read count table for annotated transcripts and TEs in the reference human genome. The resultant 56 tables were then combined in a data frame (each column for one sample) for the subsequent analysis. The gene expression similarity among all 56 samples was determined based on their gene expression profiles to verify the validity of the incorporation of the SRP193919 data. To mitigate the effects of low read counts on the similarity analysis results, rows in the resultant data frame were kept when at least 3 samples each had a count number greater than or equal to 10 to obtain a filtered data frame. The variance stabilizing transformation (VST) implemented in DESeq2^7^ was applied to this filtered data frame before similarities in expression profiles among individual samples were determined with the dist function (Euclidean distance) in R, and visualized with the R package pheatmap (Version: 1.0.12, <https://cran.r-project.org/web/packages/pheatmap/index.html>)^8^. Since we are only comparing expression levels of the same gene and TE between tumor samples and samples of normal muscle tissue adjacent to osteosarcoma, the sizeFactor reported from the VST was used to obtain the normalized expression level of transcript isoform and TE by dividing read counts in each sample from the original data frame by its corresponding sizeFactor.

## Validation for characteristics of LINE-1 mediated TE insertions

The pyliftover (<https://pypi.org/project/pyliftover/>) was used to covert TE insertion sites from hg19 to hg38. The characteristics associated with LINE-1 mediated TE insertions considered in this study include the target site duplication (TSD) length and genomic context of TE insertion preference. Specifically, the TSD sequence associated with LINE-1 mediated TE insertions, and the base composition of 50 bps up and downstream of identified insertion sites (points) (namely +/-50 bps flanking insertion sites) was analyzed and compared for both OS patients and 1KGP (using the hg38 reference genome). To understand TE insertions in the context of different genomic regions, hg38 annotations in bed format including 2000 bp upstream of the whole gene (designated as the promoters), 5'UTRs, coding regions (CDS), introns, and 3'UTRs were downloaded from UCSC Table Browser^6^ and merged by using mergeBed module implemented in BEDTools^9^. The regions that complement these merged regions within the hg38 genomic coordinates were defined as intergenic regions, which were obtained by using the complementBed module implemented in BEDTools^9^. The intersect module implemented in BEDTools^9^ was used to annotate genomic regions of LINE-1 mediated TE insertions identified in OS patients, and the ones reported in 1KGP. For TE insertions that can be identified in multiple genomic regions due to the overlapping nature of genomic annotations, the CDS was prioritized followed by the promoter, 5’UTR, 3’UTR, intron, and intergenic regions. To see the preference of LINE-1 mediated TE insertions in terms of gene length, the length (chromEnd column – chromStart column) distribution of all genes (including ones on autosomes and sex chromosomes) in the human genome hg38 (GENECODE V36 of knownGene downloaded from UCSC table browser) were stratified by TE insertion status (insertion vs non-insertion) for both OS and 1KGP data. Furthermore, the common fragile sites (CFS) of chromosomes have been associated with TE insertions in various cancers^10–12^. To explore the extent of overlaps between the CFS and LINE-1 mediated TE insertions, the CFS annotation was obtained from <https://webs.iiitd.edu.in/raghava/humcfs/>^13^ and BEDTools^9^ was used to identify their overlaps. R package plotKaryotype (Version: 1.18.0, <http://bioconductor.org/packages/release/bioc/html/karyoploteR.html>)^14^ was used to visualize these overlaps with a window size of 1 Mb.

## Identifications of OS patient-specific TE insertions

The location of the precise TE insertion at single base-pair resolution with TE detection methods based on the next-generation sequence is still difficult to determine. To identify OS patient-specific TE insertions, different cutoff values for the distance (*i.e*., 0 bp to 100 bp increased by 10 bp) between LINE-1 mediated TE insertion sites identified in OS patients and insertion sites of the same type of TE reported in 1KGP insertions were used for filtering. For each different cutoff distance, insertions were considered the same between OS patients and 1KGP if the insertions were within that distance. For each different cutoff distance considered, the resulting total number of potentially OS patient-specific TE insertions was obtained and was used to select the optimal cutoff distance to eliminate as many potentially polymorphic TE insertions identified in OS patients as possible while retaining true OS patient-specific TE insertions. To ascertain the identified OS patient-specific TE insertions as true TE insertions, IGV^15^ was used to visually validate all corresponding TE insertions. The paired reads sequenced from the TE insertion site can come with different configurations, some of which could help to manually validate TE insertions detected by different tools as shown in Supplementary Fig. S7 and described in the main text for the illustration of Fig. 4 c and d. Specifically, the following criteria were used for visual validation. (1) each insertion site identified as a valid OS patient-specific TE insertion needs to be supported by at least 1 splitting read or 3 + 3 discordant reads spanning the insertion site^12^, and (2) the paired-mates corresponding to the splitting reads or discordant reads also need to be mapped to the subfamily of either LINE-1, Alu, SVA or HERV-K with minimal mismatches based on the repeat masker annotation track in IGV. The OS patient-specific TE insertions were considered true if both criteria were satisfied. For paired mates that can be mapped to multiple TE subfamilies, the subfamily with the largest number of mapped paired mates was chosen to represent the potential subfamily associated with a given TE insertion.

## Identifications of germline and somatic TE insertions

For each patient, germline TE insertions are the same type of TE insertions observed in both the tumor sample and the paired normal sample if they were within 100 bp from each other. The normal-specific TE insertions for an individual patient are TE insertions that exclusively occurred in the normal sample (*i.e*., the same type of TE insertion does not exist within 100 bp of the insertion site in the matched tumor sample). The tumor-specific TE insertions identified for an individual patient are TE insertions that exclusively occurred in their tumor sample (*i.e*., the same type of TE insertion does not exist within 100 bp of the insertion site in the matched normal sample). To ascertain the somatic TE insertions identified in each patient with the above criterion as true, TE insertions were additionally validated between the tumor sample and their paired normal sample by using IGV^15^. Specifically, for normal and tumor-specific insertions to be considered true, for an individual patient, splitting reads or discordant reads supporting a given insertion should only exist in either the normal sample or tumor sample, respectively. To make sure TE insertion calls are not biased by the sequencing depth in either tumor sample or paired normal sample (to mitigate the potential false-negative calls associated with the lower sequencing coverage), the paired normal and tumor sample needs to have at least 3 reads spanning a given TE insertion site to support either tumor or normal -specific TE insertion.

## DNA methylation data analysis

R package minfi (Version: 1.38.0, <http://www.bioconductor.org/packages/release/bioc/html/minfi.html>)^16^ was used for methylation analysis of OS tumor samples by following the protocol <https://www.bioconductor.org/packages/devel/workflows/vignettes/methylationArrayAnalysis/inst/doc/methylationArrayAnalysis.html>. The preprocessQuantile method was used for the normalization followed by the elimination of poor-quality data. Specifically, probes that have failed (detection p-value >= 0.01) in one or more samples were removed, resulting in an elimination of 3663 probes. 11174 probes that are on sex chromosomes were also removed to reduce the dependence on the patient’s gender difference. The dropLociWithSnps implemented in minifi were then used to remove probes that overlap with common SNPs which may affect CpG sites^16^. Finally, cross-reactive probes that can be mapped to multiple places in the genome were also removed^17^, leading to a total of 427,954 probes for downstream analysis. Beta values associated with remaining probes were obtained by using the getBeta function implemented in minifi^16^ and then combined with beta values of normal control (osteoblast cell lines) based on common probes. The beta values were then converted to M values (M value = log2(Beta value / (1 – Beta value))) for subsequent comparison and statistical analysis.

To determine the methylation level associated with LINE-1 and Alu, genomic coordinates of LINE-1 and Alu from the hg38 repeat regions were acquired by using the Data Integrator function of UCSC Genome Browser^18^ with annotation from RepeatMasker^19^. It has been recently reported that DNA methylation at the transcription start site (TSS) is associated with the transcriptional repression in cancers^20^, genomic coordinates corresponding to 500 bp up and 500 bp downstream of TSS (+/- 500 bps flanking the most 5’ end of LINE-1 and Alu) associated with LINE-1 and Alu were therefore designated as 1kb of TSS. Given the lack of TSS annotation for LINE-1 and Alu in RepeatMasker, the most 5’ end of each LINE-1 and Alu was used as a proxy for its’ TSS^21^. Since most LINE-1elements in the human genome are truncated and thus incapable of active insertions, for 1kb of TSS associated with LINE-1, only the full-length LINE-1 (>= 6 kb) was considered. BedTools^9^ was used to identify probes that intersect with these identified 1kb of TSSs (*i.e*., associated with Alu and full-length LINE-1). To simplify the analysis, the average M values for each subfamily of Alu and full-length LINE-1 were calculated and the methylation level between OS tumor samples and the normal osteoblast cell lines were then visualized with the R package pheatmap^8^. Similar to the TE expression analysis, subfamilies of LINE-1 and Alu were also grouped into different categories according to their evolutionary ages^22^, and methylation levels for each category of LINE-1 and Alu were also compared between OS tumor samples and normal osteoblast cell lines.

# Supplementary Figures

## Supplementary Fig. S1. The expression comparisons for genes of interest between OS tumor samples and samples of normal muscle tissue adjacent to osteosarcoma (normal controls)

a: The similarity among 52 OS tumor samples and 4 normal controls based on expression profiles of gene transcript isoforms and TE transcripts (T: tumor samples, N: normal controls). b: The expression comparisons of satellite repeats between OS tumor samples and normal controls. c: The expression comparisons of genes associated with the homologous recombination between OS tumor samples and normal controls. d: The expression comparisons of genes associated with the non-homologous end joining between OS tumor samples and normal controls. e: The expression comparisons of genes associated with the DNA mismatch repair between OS tumor samples and normal controls. *: p < 0.05, **: p < 0.01, ***: p < 0.001, ns: not significant.

## Supplementary Fig. S2. The comparison between TE insertions identified in OS patients and TE insertions reported in 1KGP

a: The distribution of sequencing depths based on alignment results for OS tumor samples and paired normal controls (boxplot showed the first quartile, median and third quartile). b: The distribution of the numbers of TE insertions identified in OS tumor samples and normal controls by different approaches (*i.e*., MELT^23^, Mobster^24^, and consensus approach). The number of insertions was stratified by different TEs (*i.e*., LINE-1, Alu, SVA, and HERV-K). c: The numbers of different TE insertions identified by the consensus approach in the OS tumor samples and normal controls. d: The numbers of different TE insertions reported in 1KGP.


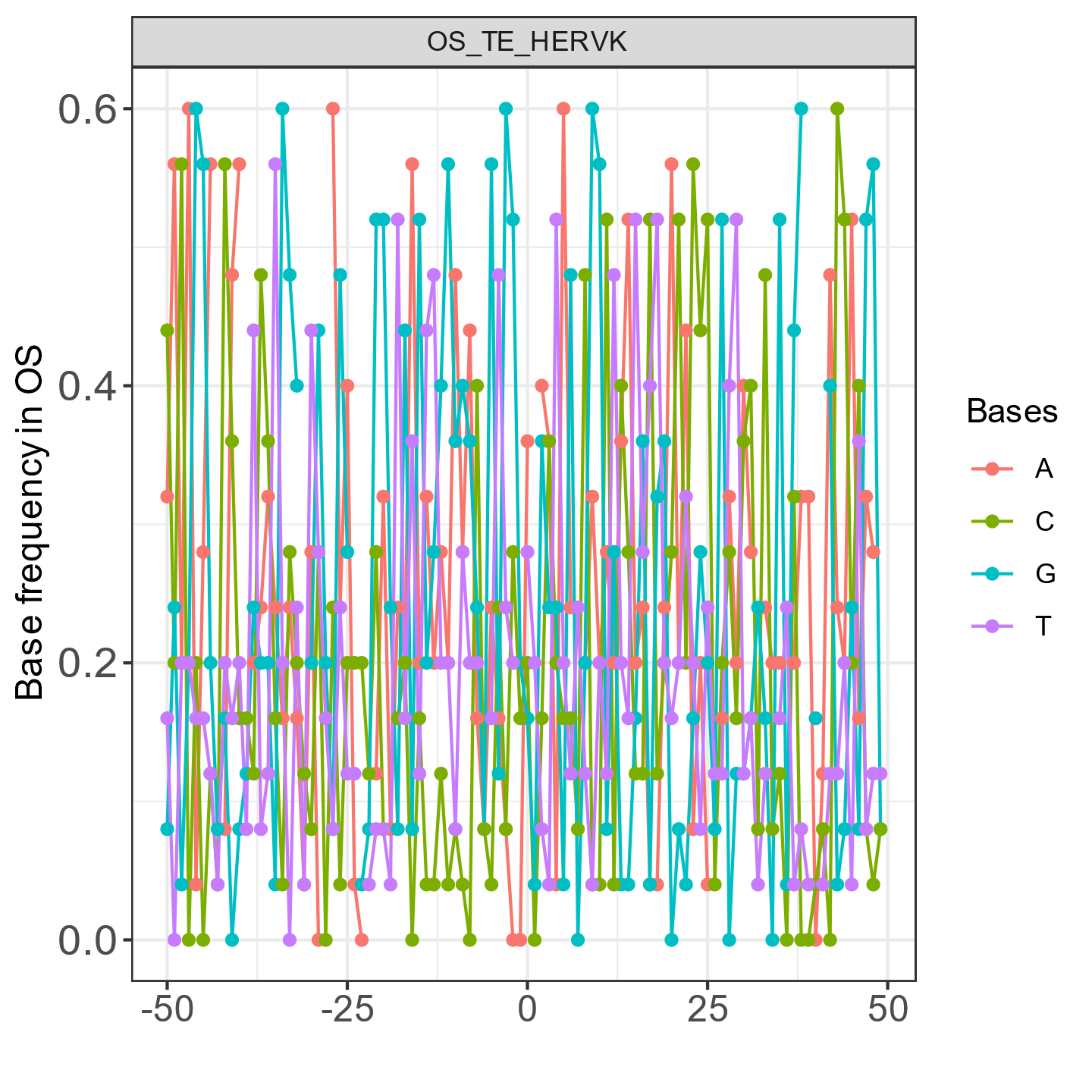


## Supplementary Fig. S3. The preference for HERV-K insertions

The nucleotide base compositions associated with HERV-K insertions identified in OS patients (+/- 50 bps flanking insertion sites marked as the zero position in the X-axis).


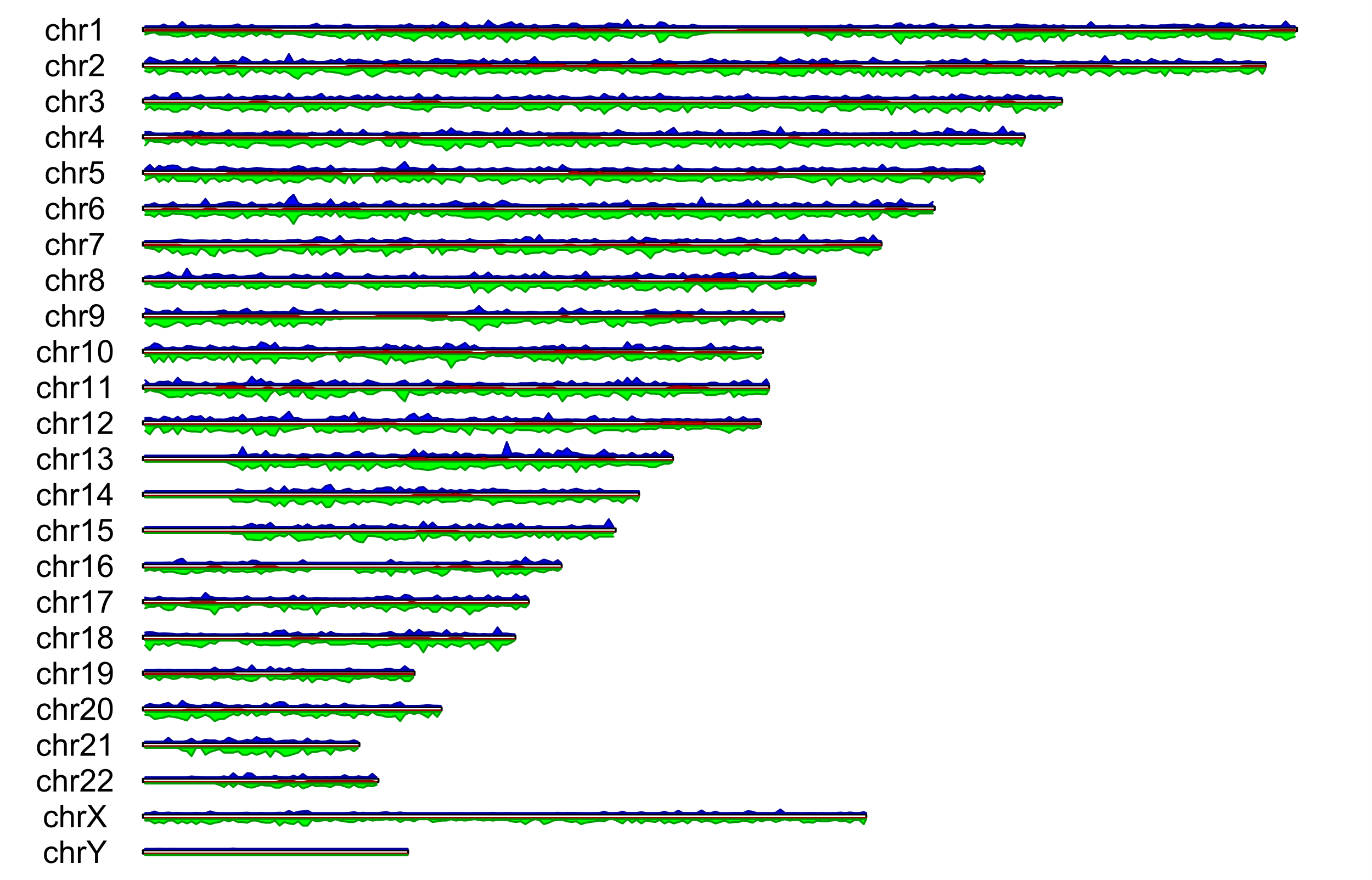


## Supplementary Fig. S4. The connection between LINE-1 mediated TE insertions and CFSs

The visualization of overlaps between LINE-1 mediated TE insertions and Common Fragile Sites (CFSs) across each chromosome in the human reference genome. The CFS sites were represented by the filled red bar (in the middle) across each chromosome. The LINE-1 mediated TE insertions identified in OS patients were represented by blue peaks above the chromosome diagram whereas LINE-1 mediated TE insertions reported in 1KGP were represented by green peaks below the chromosome diagram. A window size of 1 Mb was used for the visualization.


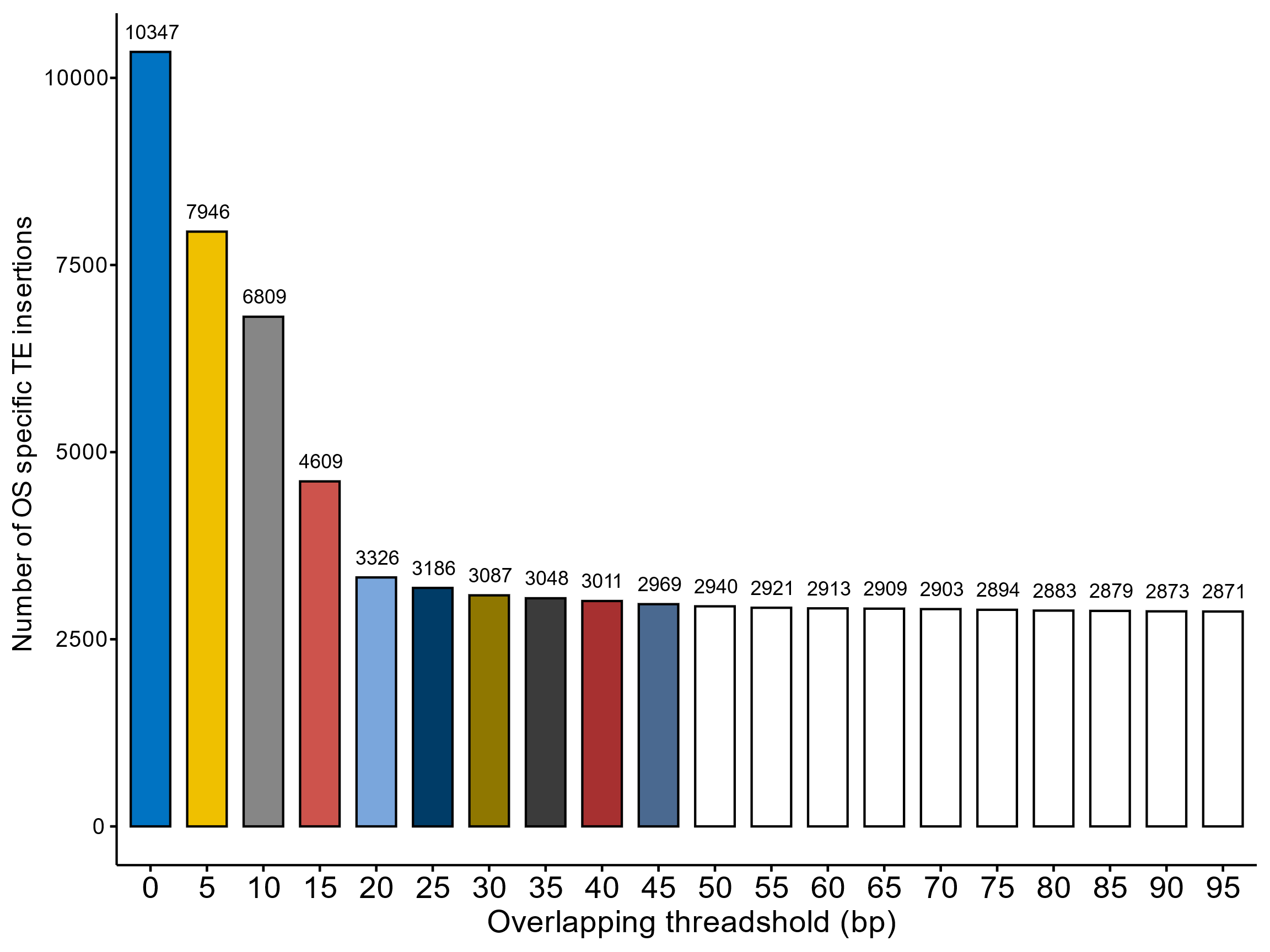


## Supplementary Fig. S5. The identification of OS patient-specific TE insertions

The number of OS patient-specific TE insertions as a function of different distances between genomic coordinates of TE insertions detected in OS patients and TE insertions reported in 1KGP for a given TE type (*i.e*., LINE-1, Alu, or SVA) for the elimination of potential polymorphic TE insertions.

## Supplementary Fig. S6. The 3,326 OS patient-specific TE insertions after filtering out polymorphic TE insertions reported in 1KGP

a: The numbers of OS patient-specific TE insertions identified in different genomic regions. b: The numbers of OS patient-specific TE insertions grouped by TE of different evolutionary ages. c: The numbers of different subfamilies of Alu associated with OS patient-specific insertions. d: The numbers of different subfamilies of LINE-1 associated with OS patient-specific insertions.


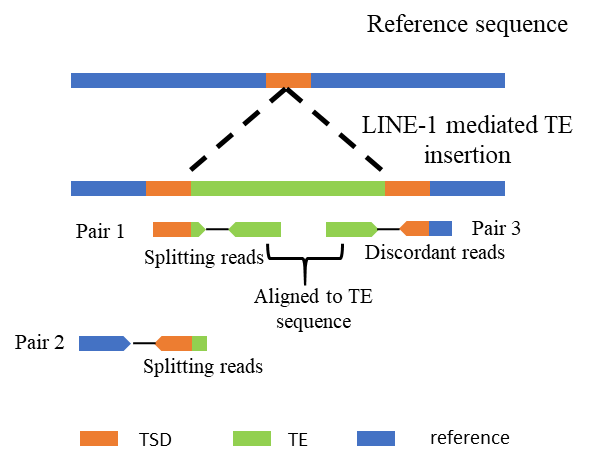


## Supplementary Fig. S7. Validation of TE insertions via IGV

The schematic description of TE insertion validation via IGV using discordant and splitting paired-end reads. There are three paired-end reads shown in this graph, marked as Pair1, Pair2, and Pair3.

## Supplementary Fig. S8. The comparison of methylation levels between OS tumor samples and normal controls

a. The comparisons of methylation levels between OS tumor samples and normal controls stratified by genomic regions mapped by different probes (log2(M/U): M value associated with each probe). b: The length distribution of Alu elements annotated in the hg 38 repeat regions (the vertical red line indicates the length of 300 bps). c: The length distribution of LINE-1 elements annotated in the hg 38 repeat regions (the vertical red line indicates the length of 6000 bps). d: The number of probes mapped to 16 different full-length LINE-1 subfamilies in the hg38 repeat regions. e: The number of probes mapped to 46 different Alu subfamilies in the hg38 repeat regions.

## Supplementary Fig. S9. Analysis workflow for the measurement of TE activities in OS patients

a: Analysis workflow for TE expression comparison between OS tumor samples and normal controls. b: Analysis workflow for TE insertion detections in OS patients. c: Analysis workflow for TE methylation comparison between OS tumor samples and normal controls.

# Supplementary Tables

All data listed in supplementary tables are represented as an Excel file titled “Supplementary tables.xlsx.”. In this excel file, the following tables are present in separate worksheets sequentially and individually.

## Supplementary Table S1: The subfamilies of major TE elements and their classifications

## Supplementary Table S2: The genes that could be potentially regulated in the host genome following TE insertions. The gene list corresponds to Supplementary Fig. S1c – e

## Supplementary Table S3: The number of insertions mapped to overlapping genomic regions

## Supplementary Table S4: The genes affected by OS patient-specific TE insertions

## Supplementary Table S5: The cancer-associated genes downloaded from public databases

## Supplementary Table S6: The 68 cancer-associated genes affected by OS patient-specific TE insertions

## Supplementary Table S7: The recurrently affected genes by germline TE insertions

# Supplementary references

1. Martin, M. Cutadapt removes adapter sequences from high-throughput sequencing reads. 3 (2011).

2. Andrews, S. *FASTQC. A quality control tool for high throughput sequence data*. (2010).

3. Dobin, A. *et al.* STAR: ultrafast universal RNA-seq aligner. *Bioinformatics* **29**, 15–21 (2013).

4. Jin, Y., Tam, O. H., Paniagua, E. & Hammell, M. TEtranscripts: a package for including transposable elements in differential expression analysis of RNA-seq datasets. *Bioinformatics* **31**, 3593–3599 (2015).

5. Jin, Y. & Hammell, M. Analysis of RNA-Seq Data Using TEtranscripts. in *Transcriptome Data Analysis* (eds. Wang, Y. & Sun, M.) vol. 1751 153–167 (Springer New York, 2018).

6. Karolchik, D. The UCSC Table Browser data retrieval tool. *Nucleic Acids Res.* **32**, 493D – 496 (2004).

7. Love, M. I., Huber, W. & Anders, S. Moderated estimation of fold change and dispersion for RNA-seq data with DESeq2. *Genome Biol.* **15**, 550 (2014).

8. Raivo Kolde. *pheatmap: Pretty heatmaps*. (2015).

9. Quinlan, A. R. & Hall, I. M. BEDTools: a flexible suite of utilities for comparing genomic features. *Bioinformatics* **26**, 841–842 (2010).

10. Fungtammasan, A., Walsh, E., Chiaromonte, F., Eckert, K. A. & Makova, K. D. A genome-wide analysis of common fragile sites: What features determine chromosomal instability in the human genome? *Genome Res.* **22**, 993–1005 (2012).

11. Helman, E. *et al.* Somatic retrotransposition in human cancer revealed by whole-genome and exome sequencing. *Genome Res.* **24**, 1053–1063 (2014).

12. Cajuso, T. *et al.* Retrotransposon insertions can initiate colorectal cancer and are associated with poor survival. *Nat. Commun.* **10**, 4022 (2019).

13. Kumar, R. *et al.* HumCFS: a database of fragile sites in human chromosomes. *BMC Genomics* **19**, 985 (2019).

14. Gel, B. & Serra, E. karyoploteR: an R/Bioconductor package to plot customizable genomes displaying arbitrary data. *Bioinformatics* **33**, 3088–3090 (2017).

15. Robinson, J. T. Integrative genomics viewer. *C O Rresp O N N Ce* **29**, 3 (2011).

16. Aryee, M. J. *et al.* Minfi: a flexible and comprehensive Bioconductor package for the analysis of Infinium DNA methylation microarrays. *Bioinformatics* **30**, 1363–1369 (2014).

17. Chen, Y. *et al.* Discovery of cross-reactive probes and polymorphic CpGs in the Illumina Infinium HumanMethylation450 microarray. *Epigenetics* **8**, 203–209 (2013).

18. Kent, W. J. *et al.* The Human Genome Browser at UCSC. 12 (2002).

19. A.F.A. Smit, R. Hubley & P. Green. *RepeatMasker Open-4.0*. (2013).

20. Ando, M. *et al.* Chromatin dysregulation and DNA methylation at transcription start sites associated with transcriptional repression in cancers. *Nat. Commun.* **10**, 2188 (2019).

21. Kong, Y. *et al.* Transposable element expression in tumors is associated with immune infiltration and increased antigenicity. *Nat. Commun.* **10**, 5228 (2019).

22. Barrow, T. M. *et al.* Analysis of retrotransposon subfamily DNA methylation reveals novel early epigenetic changes in chronic lymphocytic leukaemia. *Haematologica* haematol.2019.228478 (2020) doi:10.3324/haematol.2019.228478.

23. Gardner, E. J. *et al.* The Mobile Element Locator Tool (MELT): population-scale mobile element discovery and biology. *Genome Res.* **27**, 1916–1929 (2017).

24. Thung, D. T. *et al.* Mobster: accurate detection of mobile element insertions in next generation sequencing data. 11 (2014).
